# Mapping Brain Synergy Dysfunction in Schizophrenia: Understanding Individual Differences and Underlying Molecular Mechanisms

**DOI:** 10.1002/advs.202400929

**Published:** 2024-06-20

**Authors:** Chaoyue Ding, Ang Li, Sangma Xie, Xiaohan Tian, Kunchi Li, Lingzhong Fan, Hao Yan, Jun Chen, Yunchun Chen, Huaning Wang, Hua Guo, Yongfeng Yang, Luxian Lv, Huiling Wang, Hongxing Zhang, Lin Lu, Dai Zhang, Zhanjun Zhang, Meng Wang, Tianzi Jiang, Bing Liu

**Affiliations:** ^1^ School of Artificial Intelligence University of Chinese Academy of Sciences Beijing 100049 China; ^2^ Brainnetome Center Institute of Automation Chinese Academy of Sciences Beijing 100190 China; ^3^ State Key Laboratory of Brain and Cognitive Science Institute of Biophysics Chinese Academy of Sciences Beijing 100101 China; ^4^ School of Automation Hangzhou Dianzi University Hangzhou 310018 China; ^5^ State Key Laboratory of Cognitive Neuroscience and Learning Beijing Normal University Beijing 100875 China; ^6^ Institute of Mental Health Peking University Sixth Hospital Beijing 100191 China; ^7^ Department of Radiology Renmin Hospital of Wuhan University Wuhan 430060 China; ^8^ Department of Psychiatry Xijing Hospital The Fourth Military Medical University Xi'an 710032 China; ^9^ Zhumadian Psychiatric Hospital Zhumadian 463000 China; ^10^ Department of Psychiatry Henan Mental Hospital The Second Affiliated Hospital of Xinxiang Medical University Xinxiang 453002 China; ^11^ Department of Psychiatry Renmin Hospital of Wuhan University Wuhan 430060 China

**Keywords:** hierarchical Bayesian model, information interactions, latent factors, neurodynamics, schizophrenia, synergy dysfunction

## Abstract

To elucidate the brain‐wide information interactions that vary and contribute to individual differences in schizophrenia (SCZ), an information‐resolved method is employed to construct individual synergistic and redundant interaction matrices based on regional pairwise BOLD time‐series from 538 SCZ and 540 normal controls (NC). This analysis reveals a stable pattern of regionally‐specific synergy dysfunction in SCZ. Furthermore, a hierarchical Bayesian model is applied to deconstruct the patterns of whole‐brain synergy dysfunction into three latent factors that explain symptom heterogeneity in SCZ. Factor 1 exhibits a significant positive correlation with Positive and Negative Syndrome Scale (PANSS) positive scores, while factor 3 demonstrates significant negative correlations with PANSS negative and general scores. By integrating the neuroimaging data with normative gene expression information, this study identifies that each of these three factors corresponded to a subset of the SCZ risk gene set. Finally, by combining data from NeuroSynth and open molecular imaging sources, along with a spatially heterogeneous mean‐field model, this study delineates three SCZ synergy factors corresponding to distinct symptom profiles and implicating unique cognitive, neurodynamic, and neurobiological mechanisms.

## Graphical abstract

This study reveals significant and stable synergy dysfunction in schizophrenia (SCZ) from the perspective of dynamic information processing, and delineates three synergy factors that underlie individual variations in SCZ. Each factor displays unique patterns of atypical synergy dysfunction, aligns with specific symptom profiles, and points to distinct cognitive, neurodynamic, and neurobiological mechanisms.

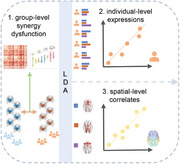

## 1 Introduction

Schizophrenia (SCZ) is a common and severe mental disorder that ranks among the top contributors to global disease burden. Accurate diagnosis and treatment are challenging, primarily because of the intricate and unclear pathological mechanisms underlying SCZ. Numerous fMRI studies have reported a range of abnormalities in brain function associated with SCZ, including disruptions in brain activity and connectivity.^[^ ^1^ ^]^ These studies have further revealed potential neuroimaging biomarkers with distinctive features related to abnormal brain function or neural circuits.^[^ ^2^ ^]^ Nevertheless, significant obstacles arise owing to the exceptionally complex nature of the brain within the context of SCZ, as well as the extensive heterogeneity observed among individuals.^[^ ^3^ ^]^ As such, it is imperative to elucidate the intricate functional organization of the brain in SCZ and untangle the heterogeneity of patients in order to gain deeper insight into the pathophysiology of SCZ and provide evidence to develop precise diagnostic and treatment strategies in the future.

The disrupted functional organization of the brain in SCZ has been widely supported by previous fMRI studies, where SCZ is often conceptualized as a “disconnection syndrome”, characterized by perturbed information interactions among brain regions.^[^ ^4^ ^]^ Most previous studies utilized the conventional metrics of functional connectivity, which quantify the similarity between regional activity, to characterize brain‐wide information interactions.^[^ ^5^ ^]^ Given that the human brain operates as a complex distributed interaction system, gaining a more precise description or measurement of its information‐processing architecture might present a new avenue for understanding the disordered organization of brain function in SCZ.

Neural information dynamics is governed by several fundamentally distinct types of information, each offering unique advantages. Taking “split‐brain” as an example,^[^ ^6^ ^]^ information that can be independently received and processed by both hemispheres (such as visual information) is redundant between the hemispheres. Information that can only be processed by one hemisphere (e.g., verbal output controlled by the left hemisphere) is unique to that hemisphere. In contrast, information for which the patient loses processing ability (such as certain aspects of intelligence) can be referred to as controlled by a synergy between the hemispheres. Generally, redundant (or shared) information endows a system with robustness; unique information promotes modularity and improves efficiency, while synergistic information facilitates functional integration into the brain. A recent breakthrough in Integrated Information Decomposition,^[^ ^7^, ^8^ ^]^ which provides an information‐resolved framework for dynamic interaction systems by combining methods of Information Decomposition and Integrated Information, has the potential to unravel the different forms of information interactions disrupted in the SCZ brain from the perspective of neural information dynamics.

After gaining a comprehensive understanding of the patterns of brain information processing impairment in SCZ, it is important to recognize the substantial heterogeneity present among patients with SCZ across diverse facets, including brain atypicality,^[^ ^9^ ^]^ core SCZ symptoms,^[^ ^10^ ^]^ cognitive aptitude,^[^ ^11^ ^]^ and antipsychotic responses.^[^ ^12^ ^]^ Notably, recent neuroimaging‐clinic correlation analyses have delved into the neurobiological foundation of phenotypic diversity in SCZ, revealing that delineating anatomically‐defined subgroups can enhance the accuracy of predicting SCZ symptom severity,^[^ ^10^, ^13^ ^]^ and that two disease‐progression‐modeled atrophy trajectories relate to different antipsychotics‐only effects.^[^ ^12^ ^]^ Many of the prior SCZ subtyping studies have assumed that each participant belonged to a distinct categorical subtype. However, multiple symptom domains manifest as continuous variations across individuals,^[^ ^14^ ^]^ leading to high variance, even within subtypes.^[^ ^12^, ^15^ ^]^ Hence, in light of the notion that interindividual variability in SCZ likely mirrors varying degrees of factor expression and related mechanisms, it becomes intriguing to search for the multidimensional pathological mechanisms coexisting in individuals with SCZ, and their associations with symptoms through a systematic characterization of dysfunctional organization. Latent Dirichlet Allocation (LDA),^[^ ^16^ ^]^ a variant of the Bayesian models which can automatically unearth statistical co‐occurrence patterns and offer multidimensional factor expressions, has been successfully deployed to identify hidden atrophy factors underlying Alzheimer's disease and hidden components underlying cognition.^[^ ^17^, ^18^ ^]^ As such, uncovering the individual differences of abnormal brain information interactions among individuals with SCZ from a multidimensional perspective, may facilitate a more profound understanding of the biological heterogeneity and pathological mechanisms underlying SCZ.

To reveal abnormal brain functional organization and untangle its heterogeneity in individuals with SCZ, we attempted to address three progressively unfolding questions: Do decomposed information components capture dysfunctional organization in the SCZ?; Are multidimensional dysfunctional patterns present in SCZ?; and How do the distinct genetic, cognitive, molecular, and neurodynamic mechanisms and symptoms relate to each dysfunctional pattern? Utilizing an information‐resolved approach in a multicenter fMRI dataset including 538 patients with SCZ and 540 normal controls (NC), we first investigated the synergistic and redundant brain information interactions in each individual, revealing a significant and consistent discrepancy in synergistic interactions between the two groups. We subsequently applied a hierarchical Bayesian model (LDA) to decompose whole‐brain synergy dysfunction patterns in individuals with SCZ into three latent factors that explained symptom heterogeneity. Using extensive imaging transcriptomic analysis by integrating factor patterns with normative gene expression data from the Allen Human Brain Atlas,^[^ ^19^ ^]^ we further found that each factor corresponded to a portion of the SCZ risk gene set, and revealed the molecular organization and neurobiological underpinnings of each factor. Combining data from NeuroSynth and open molecular imaging sources, along with a spatially heterogeneous mean‐field model (pMFM),^[^ ^20^ ^]^ we further demonstrated the distinct cognitive, molecular, and neurodynamic correlates of each factor.

## 2 Results

### 2.1 Whole‐Brain Synergy Dysfunction in SCZ

**Figure 1:**
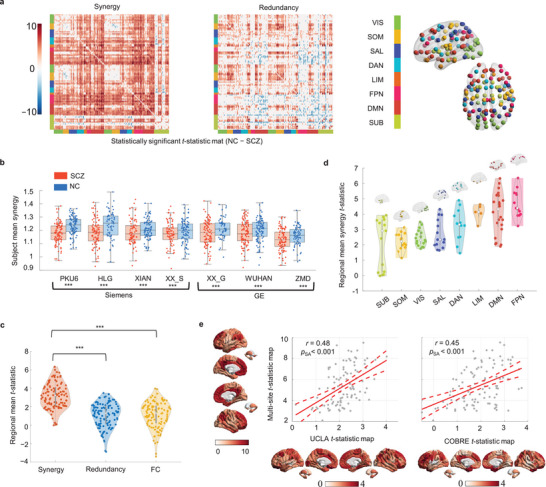
Dysfunction of synergy networks in SCZ. a) Statistically significant *t*‐statistic matrices displaying between‐group differences in synergistic and redundant interactions (NC−SCZ; 538 SCZ and 540 NC; unpaired two‐sided *t*‐test). The large *t*‐statistic values for synergistic interactions indicate significant reduction in synergy of the SCZ group. b) Scatter‐box chart comparing mean synergy between SCZ and NC groups across different sites. From left: PKU6 (*p* < 2.5 × 10^−6^), HLG (*p* < 5.9 × 10^−4^), XIAN (*p* < 2.5 × 10^−3^), XX_S (*p* < 1.2 × 10^−2^), XX_G (*p* < 7.9 × 10^−3^), WUHAN (*p* < 4.2 × 10^−3^), and ZMD (*p* < 7.9 × 10^−3^), unpaired two‐sided *t*‐test. Each colored circle represents one subject. The upper and lower boundaries of the box represent the first and third quartiles, while the median is indicated by the horizontal line inside the box. The lower and upper whiskers extend to 1.5 times the interquartile range. Synergy network dysfunction in the SCZ group was observed at all independent sites, despite the use of different scanners. c) Regional mean *t*‐statistic values for three types of interactions (synergy: mean = 3.83, s.d. = 1.8; redundancy: mean = 1.33, s.d. = 1.9; functional connection: mean = 1.13, s.d. = 1.9). *** denotes *p* < 0.001; unpaired two‐sided *t*‐test. d) Distinct resting‐state network profiles for regional synergy dysfunction. Each violin plot illustrates the synergy *t*‐statistic distribution of brain regions assigned to the indicated subnetwork on the *x*‐axis. The *t*‐statistic values of eight canonical resting‐state networks increase from low‐order sensorimotor cortices (VIS, SOM) to high‐order association cortices (FPN, DMN). e) Robustness of synergy *t*‐statistic map across different SCZ datasets. Significant correlations were observed between the results of multi‐site dataset and UCLA/COBRE datasets (UCLA, *r* = 0.48, *p*_SA_ < 0.001; COBRE, *r* = 0.45, *p*_SA_ < 0.001), indicating that the robustness of synergy dysfunction findings. Correlation analyses were performed using Pearson's correlation. *p* values were estimated using spatial autocorrelation (SA) preserving surrogate maps generated by BrainSMASH method.^[^ ^32^ ^]^

To explore variations in information‐based interactions among distinct brain regions in individuals with SCZ, we applied a well‐established Integrated Information Decomposition method to dissect resting‐state fMRI data derived from a multi‐site cohort.^[^ ^7^ ^]^ This dataset comprised fMRI data of 538 individuals with SCZ and 540 age‐ and gender‐matched NC collected from six Chinese hospitals using seven different MRI scanners. Through the implementation of the information‐resolved approach, the preprocessed fMRI signals for each pair of ‘Schaefer‐115′ regions were deconstructed, revealing the distinct synergistic and redundant interactions specific to their respective cerebral regions. We further conducted a comparative analysis between the SCZ and NC groups, comparing the two information components, by employing a two‐sample *t*‐test while controlling for age, gender, and site. This analysis yielded two symmetrical matrices (115×115) of the intergroup differences (contrast = NC−SCZ). There was a pronounced reduction in synergistic interactions across the majority of cerebral regions in individuals with SCZ, whereas redundant components exhibited both increases and decreases across diverse brain regions (**Figure** **1**a).

At the whole‐brain level, by averaging the synergy scores across the whole brain (6555 region pairs, the lower triangular portion of the 115 × 115 matrix), and conducting between‐group comparisons for each research site, we observed that the whole‐brain mean synergy scores within the SCZ group were significantly lower than those in the NC group across all seven scanners, including both Siemens and GE scanners (Figure 1b, two‐sample *t*‐test, *p* < 0.05). However, using a similar analysis, we did not detect any significant between‐group differences in whole‐brain mean redundancy or FC scores across all research sites (Figure S1, Supporting Information).

At the brain‐region level, we computed the row‐wise or column‐wise averages of the between‐group difference matrices (i.e., *t*‐statistical matrices from Figure 1a), and derived the mean regional disparities for the two information components. This measurement quantified the average distinctions in information interactions for each brain region, resulting in 115 scores for each information component. We conducted the same between‐group comparisons for FC and calculated the mean regional FC disparities based on the same samples to compare to the two information components. As shown in Figure 1c, the synergy scores exhibited significantly greater disparities than redundancy (paired sample *t*‐test: *t* = 11.8, *p* < 0.001) and FC (*t* = 16.1, *p* < 0.001). Concerning the mean regional *t*‐statistical values, there were no intergroup differences between redundancy and FC (*t* = 1.59, *p* = 0.11), further suggesting a similarity in brain organization between redundancy and FC.^[^ ^8^ ^]^ When summarizing regional synergy *t*‐statistic values using classical resting‐state networks,^[^ ^21^ ^]^ we observed a gradual increase in synergy disparity across the cortical hierarchy, spanning from primary somatomotor (SOM) and visual (VIS) networks to higher‐order default mode (DMN) and frontoparietal (FPN) executive control networks (Figure 1d).

Considering the evidence at both the whole‐brain (Figure 1b) and brain‐region (notably the DMN and FPN, Figure 1d) levels, we observed that abnormalities in synergistic interactions were more pronounced and consistent in individuals with SCZ. Hence, our subsequent research focused primarily on the synergy component. We directly compared the synergy patterns between the SCZ and NC groups at three levels: connection matrices, brain regions, and functional networks (Figure S2, Supporting Information). In the NC group, we observed a network‐specific gradient in synergistic interactions among networks, with the FPN and DMN (higher‐order association networks; modular integrated networks) exhibiting significantly higher synergy levels, and the SOM network (primary motor cortex; modular dissociative network) exhibiting significantly lower synergy levels than the whole‐brain average. This network‐specific gradient aligns with the definition of synergy, wherein two brain regions depend on each other to generate new information. However, in the SCZ group, this network‐specific gradient faded away, particularly in the SOM and DMN, underscoring the importance of examining synergy dysfunction from a whole‐brain perspective.

Notably, we replicated our analyses in two additional independent datasets, COBRE (SCZ/NC, *n* = 70/70) and UCLA (SCZ/NC, *n* = 47/115),^[^ ^22^, ^23^ ^]^ using identical statistical procedures. By comparing the similarity, measured with Pearson's correlation, between the regional *t*‐statistic values for synergy in these datasets and the corresponding distributions from our multi‐site primary dataset, we observed a highly reproducible aberrant synergy pattern in SCZ (Figure 1e and *r* = 0.45, *p*_SA_ < 0.001 between COBRE and multi‐site datasets; *r* = 0.48, *p*_SA_ < 0.001 between UCLA and multi‐site datasets). Further, when utilizing different brain parcellation atlases, including the Brainnetome atlas (246 regions),^[^ ^24^ ^]^ and the Desikan‐Killiany atlas (83 regions),^[^ ^25^ ^]^ we observed that the aberrant synergy patterns in SCZ remained stable at both the whole‐brain and brain‐region levels (Figure S3, Supporting Information). Taken together, our findings revealed a widespread reduction in synergistic interactions throughout the whole brain in subjects with SCZ compared to NC. This dysfunctional pattern exhibits high reproducibility and robustness, making it a potential hallmark for characterizing the pathological mechanisms underlying SCZ.

### 2.2 Three SCZ Synergy Factors with Distinct Interaction Patterns Inferred by LDA Model

Although we detected a pervasive reduction in synergistic interactions in SCZ, it was crucial to acknowledge the substantial heterogeneity at the individual level. Whether at the brain‐region level, brain‐network level, whole‐brain level, or cortical level, the variance in the distribution of synergistic interactions for SCZ was significantly greater than that for NC (Figure S4, Supporting Information). Given that heterogeneity poses a critical challenge in unraveling the pathological mechanisms underlying SCZ,^[^ ^14^ ^]^ it emphasizes the importance of focusing on dissecting the heterogeneity of abnormal synergistic interaction patterns in SCZ. Additionally, by recognizing the inherent continuous variation among individuals with SCZ, we utilized a dimensional Bayesian approach (LDA) to dissect the synergy patterns in SCZ into distinct abnormal modes, referred to as factors.^[^ ^16^ ^]^ This decomposition framework harmonizes categorical and dimensional models, enabling individuals with SCZ to express multiple factors simultaneously, thus better accommodating the need to dissect the synergistic heterogeneity in SCZ.

**Figure 2:**
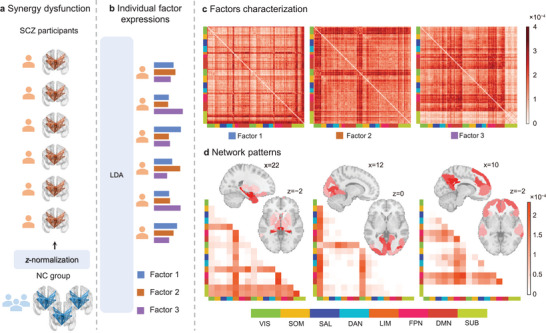
Three synergy factors with distinct interaction patterns inferred by LDA model.^[^ ^16^ ^]^ a) Whole‐brain synergistic interactions of both groups and normalization for SCZ. Synergy matrices for each individual with SCZ were normalized against the corresponding distribution in NC using *z*‐scores. b) Inference of three overarching synergy factors using LDA model. The model enables the extraction of latent factors characterized by mixed membership, based on the assumption that multiple latent factors exist in subjects with SCZ. Consequently, each participant's network organization of synergistic interactions was modeled as simultaneously influenced by multiple latent synergy factors. c) Factor‐specific synergistic interaction patterns. LDA model simultaneously estimated the corresponding synergy patterns associated with each factor, referred to as factor‐specific synergy patterns. d) Network‐level synergy patterns of three latent factors, computed by averaging the values of synergistic interactions within functional subnetworks.

In the primary multisite dataset, we initially conducted a *z*‐transformation on the synergy matrices (115×115) for each subject with SCZ relative to the NC group at each matrix position (**Figure** **2**a). The LDA method was subsequently applied to the *z*‐scored synergy matrices in the SCZ (Figure 2b). This model requires the predefinition of a hyperparameter, namely, the number of factors. We experimented with factor counts of 2, 3, and 4 to assess the stability of the model, finding that the model produced the best results when using three factors (Figure S5, Supporting Information). To further assess the robustness of the 3‐factor solution, we divided the multisite primary dataset into two sub‐datasets based on the MRI scanner type: SCZ_S (Siemens) and SCZ_G (GE) (Table S2, Supporting Information). We further conducted LDA decomposition separately on these two sub‐datasets, finding a high degree of consistency between the 3 factors obtained in the two sub‐datasets and those derived from our main results (*r* ≥ 0.85). The 3 factors obtained from the two sub‐datasets were also found to be highly correlated (*r* ≥ 0.72) (Table S3, Supporting Information). Moreover, replicating the entire process in the COBRE and UCLA datasets, we obtained three factors representing SCZ synergy abnormalities that exhibited consistency with our main findings (COBRE: *r* ≥ 0.38, UCLA: *r* ≥ 0.42) (Table S4, Supporting Information).

Each factor potentially characterized a specific synergy dysfunction pattern among the 115 cortical and subcortical regions in SCZ (Figure 2c,d). Factor 1 primarily involved the subcortical and limbic networks, both of which exhibited synergy abnormalities relative to other brain regions across the whole brain. It is also worth noting that the focus of factor 1 on the subcortical regions was uneven, primarily concentrating on the amygdala, accumbens, hippocampus, and thalamus, rather than the striatum. Factor 2 was associated with abnormal synergistic interactions between the visual cortex and other brain networks. Factor 3 was mainly linked to abnormal synergy patterns within the cortex, particularly encompassing the DMN and FPN, along with their interactions with networks such as SOM and salience/ventral attention (SAL). Collectively, we decomposed the heterogeneous synergy patterns in SCZ into three robust and dissociable abnormal modes (factors), each characterized by different networks.

### 2.3 Individual‐Level Associations Between Synergy Factor Expressions and SCZ Participants’ Characteristics

**Figure 3:**
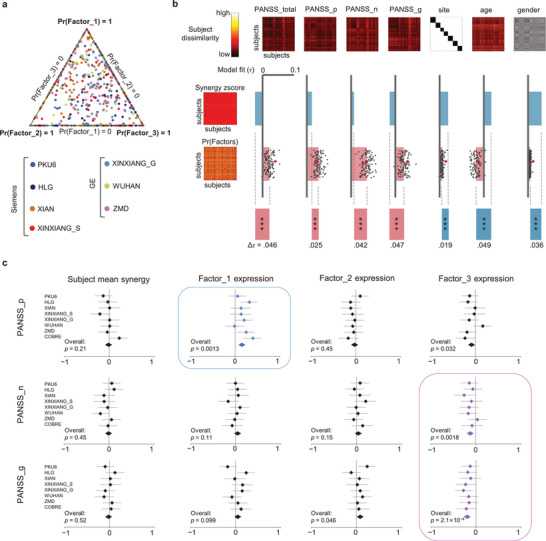
Factor compositions and associations with participants’ characteristics. a) Factor compositions of SCZ in the multi‐site samples. Each dot corresponds to a participant, and its location in barycentric coordinates indicates this participant's factor composition. The corners of the triangle represent pure factors, and dots closer to the corners indicate a higher probability of the corresponding factor. Dots along the edge signify the co‐expression of two factors. b) Dissimilarity matrices of synergy *z*‐score and factors probability compared with dissimilarity based on different participants’ characteristics. Subject dissimilarity matrices were calculated using the Euclidean distance of variables between participants. Model fit was assessed using the Kendall rank correlation coefficient (Kendall *τ*). Red diamond represents the final estimate out of 100 random estimates. It was evident that the subject dissimilarity matrix obtained from factor expression loadings shows a significantly higher correlation with symptoms (PANSS), while being significantly less associated with variables (gender, age, site) common to both NC and SCZ participants. This highlighted the effectiveness of LDA in identifying symptom dimensions in the synergy interactions of SCZ. c) Forest plots for correlations of three PANSS subscale scores and three factor expression loadings, compared with the subject mean synergy. All the seven sites and COBRE dataset are included in each plot. The Pearson's correlations and their 95% confidence interval (CI) were reported. An overall correlation was calculated by merging all participants together. Significant overall correlations after FDR multiple comparison correction are highlighted with blue and purple boxes. The results indicate that factor 1 was correlated with positive PANSS subscale, while factor 3 was correlated with negative and general PANSS subscales.

In the primary multi‐site dataset, most individuals with SCZ expressed multiple synergy factors, rather than a single one (**Figure** **3**a). Moreover, the distribution of factor composition among individuals with SCZ across different sites remained similar, indicating the independence of factor expression from site‐specific variations. We subsequently explored the associations between latent synergy factors expressed by patients with SCZ and their clinical symptoms while considering other non‐clinical variables by employing the representational similarity analysis (RSA) method.^[^ ^26^ ^]^ Specifically, we calculated the corresponding dissimilarity matrices (538×538) among SCZ in the primary multi‐site dataset based on Euclidean distance for factor expression, original synergy *z*‐scores, Positive And Negative Symptom Scale (PANSS) total and subscale scores, as well as age, gender, and site. To mitigate bias introduced by the initialization of the Bayesian decomposition algorithm, we conducted 100 iterations of synergy factor decomposition. We separately computed the Kendall rank correlation (Kendall *τ*) between the lower triangles of the dissimilarity matrices for the factor expression loadings (and original synergy *z*‐scores as a control analysis) and the dissimilarity matrices related to clinical and non‐clinical characteristics. In doing so, we found that the factor expressions were significantly correlated with the PANSS total score and its three subscale scores, and that the original synergy *z*‐scores primarily captured non‐clinical variables, such as age, gender, and site (*p* < 0.05) (Figure 3b). By employing general linear regression models to establish connections between the expression of the three factors and non‐clinical characteristics, we confirmed that the factor expression loadings were unrelated to variables such as age, gender, education length, and head motion (Figure S6, Supporting Information).

To further explore the specific associations between each factor and the clinical symptoms, we separately calculated the relationships (Pearson's correlation) between the expression loadings of the three factors and the three subscales of the PANSS within each site. Analysis was further conducted using the COBRE dataset. We obtained an overall correlation effect by aggregating the multisite and COBRE datasets, observing a significant positive correlation between factor 1 and positive symptoms (overall: *r* = 0.14, *p* = 0.0013), while factor 3 exhibited a significant negative correlation with negative symptoms (overall: *r* = −0.14, *p* = 0.0018) and general psychopathology symptoms (overall: *r* = −0.15, *p* = 2.1 × 10^−4^). Factor 2 and the original mean synergy score were not significantly correlated with the three symptom dimensions (*p* > 0.05, FDR‐corrected) (Figure 3c). Moreover, upon separately correlating the expression loadings of the three factors with all 30 specific PANSS symptoms (using Pearson's correlation), we consistently found that factor 1 expression loading had the highest number of significant correlations with positive symptom items, whereas factor 3 expression loading displayed the most substantial correlations with negative and general psychopathology symptom items (Figure S7, Supporting Information). The correlation results with the 30 PANSS symptom items were replicable in the two sub‐datasets categorized by MRI scanner type: SCZ_S (Siemens) and SCZ_G (GE) (Figure S8, Supporting Information). To evaluate whether factor expression could serve as a basis for subtyping, we stratified each patient with SCZ into a corresponding subgroup according to the highest expression of synergy factors (Figure S9, Supporting Information). As anticipated, the factor 1 subgroup exhibited significantly higher PANSS positive scores, whereas the factor 3 subgroup exhibited lower PANSS negative and general scores. Furthermore, the three subgroups demonstrated a network specificity similar to that of the original factors in synergy as compared to the NC group.

Next, we assessed the potential contributions of medication and disease chronicity to the identified synergy factors. First, we confirmed that there was no significant correlation after FDR correction between the three factor expressions and antipsychotic dose (chlorpromazine equivalents at the scanning, CPZ‐eq; factor 1: *r* = 0.091, *p* = 0.14; factor 2: *r* = −0.047, *p* = 0.32; factor 3: *r* = −0.044, *p* = 0.24; Figure S10, Supporting Information), and illness duration (factor 1: *r* = −0.069, *p* = 0.30; factor 2: *r* = 0.045, *p* = 0.45; factor 3: *r* = −0.028, *p* = 0.50; Figure S11, Supporting Information). Subsequently, we regressed out CPZ‐eq and illness duration from each synergistic interaction for the synergy matrix of the medicated SCZ group, and re‐decomposed the factors. The three factor patterns obtained from the results remained stable (mean correlation with CPZ‐eq regressed out: *r* = 0.968; illness duration regressed out: *r* = 0.961). Based on these results, it appears that these symptom‐related factors are relatively independent of antipsychotic medication and illness duration.

Considering that physiological disparities in SCZ may also affect function,^[^ ^27^ ^]^ we delved deeper into the relationship between the synergy factors and total gray matter volume (GMV, 344 744 voxels; Figure S12, Supporting Information). No significant correlation between total GMV and three factor expressions for the SCZ group was observed after FDR correction for multiple comparisons (factor 1: *r* = −0.01, *p* = 0.82; factor 2: *r* = −0.078, *p* = 0.12; factor 3: *r* = 0.09, *p* = 0.12). After regressing out the total GMV from each synergistic interaction for the synergy matrix of the SCZ group, and re‐decomposing the factors, the three factor patterns remained stable (mean correlation: *r* = 0.976). These results indicated that the synergy factors were not driven by brain structure.

In summary, we found that the three factors expressed by SCZ patients demonstrated specific correlations with clinical symptoms, while remaining unrelated to other demographic and confounding variables.

### 2.4 Transcriptomic Association Analysis for Three Factors

Next, we conducted transcriptomic association analysis and gene ontology (GO) enrichment analyses to gain insight into the neurobiological mechanisms underpinning the three factors identified in our previous analysis (Figure S13a, Supporting Information). We first aggregated the matrix‐level synergy patterns into factor vectors (synergy factor maps) by summing at the regional level, denoted as “*y*.” We then mapped normative regional gene expression profiles from the Allen Human Brain Atlas (AHBA) to the functional parcellation used previously,^[^ ^19^ ^]^ generating a gene expression matrix (115 × 15633), referred to as “*X*.” Subsequently, we calculated the Pearson's correlations between *X* and *y*, selecting the top and bottom 1500 genes (≈10%) for each factor as synergy risk gene sets. Disease enrichment analysis revealed that both the top (union: *n* = 4469) and bottom (union: *n* = 4457) gene sets were significantly enriched for SCZ‐related genes (Figure S13d, Supporting Information), further supporting the notion that the three factors inferred by LDA are specific to SCZ.

Having established the specificity of the synergy risk gene sets for SCZ and considering the significant co‐expression of these genes in brain tissues, we aimed to understand whether distinct biological pathways were implicated in each factor. To achieve this, we employed a virtual gene knockout procedure based on gene co‐expression. We first investigated the three gene‐factor correlations between the co‐expression of synergy risk genes (union: *n* = 6913, Figure S13b, Supporting Information) and the synergy dysfunction pattern of each factor. Two sets of correlations (factor 1 and 3, Figure S13c, Supporting Information) achieved significance (*p*_SA_ < 0.001), indicating that these factors were subjected to the collective influence of synergy risk genes. Subsequently, the virtual gene knockout procedure, which simulates varying gene deletion effects on gene‐factor correlations, and quantifies the change in correlation coefficients before and after deletion, successfully segregated the total 6913 synergy risk genes into two sets of genes associated with each factor. The two sets for each factor, a positive‐contribution gene set (GCI^+^) and a negative‐contribution gene set (GCI^−^), exert contrasting impacts on the gene‐factor correlation, and could provide insights into the distinct pathological mechanisms underlying each factor, which could be further explored through enrichment analysis.

The top 15 terms in GO enrichment analysis of the GCI^+^ and GCI^−^ for each factor are displayed in Figure S14, Supporting Information. All three factors shared eight common items related to fundamental neuron components and functions: postsynapse (GO: 00 98794), neuron projection (GO: 00 43005), plasma membrane protein complex (GO: 00 98797), microtubule organizing center (GO: 0 005815), microtubule cytoskeleton (GO: 00 15630), ATP hydrolysis activity (GO: 00 16887), ATP‐dependent activity (GO: 014 0657), ATPase‐coupled transmembrane transporter activity (GO: 00 42626). However, overlap in the GO terms between factors 1 and 3 was minimal, suggesting that these two factors have distinct biological pathways. Factor 1 was more strongly associated with fundamental neuron anatomical entity and cellular process, including: centrosome (GO: 0 005813), neuronal cell body (GO: 00 43025), somatodendritic compartment (GO: 00 36477), catalytic complex (GO: 1 902 494), hydrolase activity, acting on acid anhydrides (GO: 00 16817), carbohydrate derivative metabolic process (GO: 1 901 135), pyrophosphatase activity (GO: 00 16462), adenyl nucleotide binding (GO: 00 30554), cyclin‐dependent protein kinase activity (GO: 00 97472). In contrast, factor 3 was specifically enriched in neurotransmitter transport and signaling transduction, such as: synapse (GO: 00 45202), synaptic membrane (GO: 00 97060), glutamatergic synapse (GO: 00 98978), potassium channel complex (GO: 00 34705), plasma membrane protein complex (GO: 00 98797), postsynaptic membrane (GO: 00 45211), and glutamate receptor activity (GO: 00 98797). Factor 2 GCI^+^ genes shared neurobiological underpinnings with factor 3 GCI^+^, while factor 2 GCI^−^ genes were similar to factor 1 GCI^−^ genes. These results indicated that factors 1 and 3 developed as the result of the most distinct pathological mechanisms, whereas factor 2 shared some similarities with factors 1 and 3.

### 2.5 Cognitive, Molecular and Neurodynamical Correlates of the Three Factors

**Figure 4:**
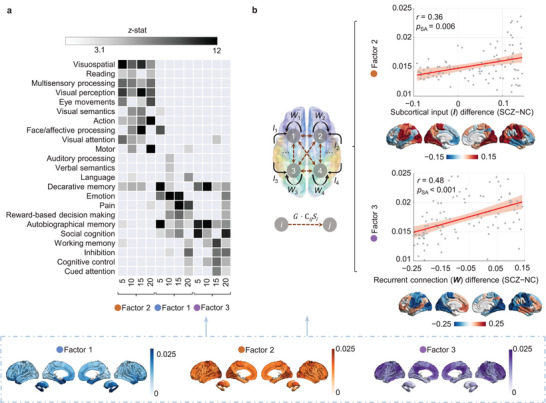
Spatial correlates of dysfunction patterns summed across brain regions in three SCZ synergy factors. a) NeuroSynth term‐based meta‐analysis.^[^ ^30^ ^]^ Each factor was characterized by the top 20% of brain regions, revealing that the three factors corresponded to a wide and diverse range of cognitive topics. b) A parameterized mean‐field model (pMFM) was employed simulate functional dynamic signals and infer regional microcircuit parameters,^[^ ^20^ ^]^ specifically recurrent connection (***W***) and subcortical input (***I***). Differences in these two microcircuit parameters between individuals with SCZ and NC were related to the three factors to explore their neurodynamic mechanisms. Significant positive linear correlations between the ***W*** difference and factor 3, as well as the ***I*** difference and factor 2 were observed. Spatial Pearson's correlations were assessed using a SA permutation test.

Cognitive impairment is a significant feature of SCZ, affecting various cognitive domains including attention, memory, reasoning, and processing speed.^[^ ^28^ ^]^ To empirically explore the relationship between the factors and different cognitive domains, we performed a lexicon‐driven meta‐analysis using NeuroSynth,^[^ ^29^, ^30^ ^]^ a frequently used tool for delineating extensive brain patterns in the context of their cognitive significance. We utilized 24 classic behavior‐ and cognition‐related topic terms from previous studies,^[^ ^30^ ^]^ covering a wide range of visual, motor, and cognitive functions. For each factor, we summed the dysfunction in synergistic interactions across each brain region and selected the top 20% of the brain regions with the most dysfunction. The results revealed that these three factors corresponded to a diverse range of cognitive topics (**Figure** **4**a). Factor 2 exhibited strong associations with visual and multisensory processing, in addition to motion. Factor 1 was primarily associated with emotions and pain. In contrast, factor 3 exerted the greatest influence on social cognition and working memory.

To complement our multi‐scale findings, we utilized a parametric mean‐field model (pMFM) to conduct biophysical computational simulations by synchronously fitting the functional connectivity and dynamics of both empirical and simulated BOLD signals.^[^ ^20^ ^]^ This modeling approach allowed us to estimate two regional microcircuit parameters (recurrent connections, ***W***; subcortical inputs, ***I***; Figure 4b) for individuals with SCZ and NC. First, we established that the model had a good fit with our data (Figure S15, Supporting Information). We then explored the neurodynamic mechanisms related to these three factors by examining between‐group differences in these microcircuit parameters. Our analysis revealed significant positive linear correlations between ***W*** difference and the factor 3, as well as ***I*** difference and the factor 2 (Figure 4b). This observation suggests that factor 2 and 3 were driven by subcortico‐cortical interactions and endogenous inputs, respectively.

We further leveraged the Neuromaps toolbox to quantitatively assess the molecular and functional enrichment of the three synergy factor maps.^[^ ^31^ ^]^ Neuromaps compiled a series of spatially comparable brain maps from previous studies and implemented robust transformations between standard coordinate systems. As shown in Figure S16 (Supporting Information), 10 of the 36 neurotransmitter transporters and receptors based on PET images were significantly associated with at least one factor. Factor 1 was positively associated with serotonin and dopamine receptors, while factor 2 negatively correlated with mu‐opioid receptor expression, and factor 3 positively correlated with cannabinoid and acetylcholine receptors. The spatial correlates of the three factors for the classic 17 maps quantifying brain structure and function with considerable profundity and detail are presented in Figure S17 (Supporting Information). The findings revealed that these three factors corresponded to a diverse and distinct set of maps compared with the initial map of synergy dysfunction. This discovery sheds further light on the heterogeneity in SCZ and offers insight into the potential mechanisms underlying its development.

The above analysis indicates that the three factors exhibited distinct associations with cognitive, molecular, and neurodynamic characteristics, and their specificity may be related to their association with psychiatric symptoms.

## 3 Discussion

This study comprised a pioneering and comprehensive investigation of information interactions in SCZ, initially decomposing the intrinsic dynamics of resting‐state BOLD signals in SCZ patients from an information‐resolved perspective, followed by dissecting the heterogeneity in the atypical patterns of brain‐wide interregional information interactions among these patients using a Bayesian dimensional technique, and ultimately revealing the genetic, cognitive, molecular, and neurodynamic mechanisms contributing to this heterogeneity through various association modeling methods.

The first major finding of our study was the consistent and reproducible pattern of synergy abnormalities in patients with SCZ. At both of the whole‐brain and brain‐region levels, synergy in patients with SCZ demonstrated a significant decrease. However, such alterations were not observed for redundancy, which represents another information‐based metric decomposed alongside synergy, and traditional FC. Particularly at the whole‐brain level, neither mean redundancy nor mean FC showed significant differences between the SCZ and NC groups. Conceptually, synergy quantifies the combination and integration of unique information from distinct regions, fundamentally representing their complementary nature. In contrast, redundancy refers to shared information provided between different brain regions, that remains equally accessible from either source.^[^ ^7^, ^8^, ^33^, ^34^ ^]^ In terms of contributions to the brain system, synergy plays a pivotal role in intricate computational processes, including cognitive functions, which cannot rely solely on shared information, but instead necessitates integration across diverse brain regions. Redundancy provides the brain with robustness, ensuring the reliability of specific survival channels such as basic sensorimotor functions. Traditional FC further measures the linear similarity between temporal fluctuations in brain regions, and is incapable of addressing higher‐order statistical phenomena. This demonstrates a significantly greater similarity with redundancy than with synergy.^[^ ^8^ ^]^ As such, the observed reduction in synergy, as opposed to redundancy and traditional FC, indicates a significant impairment in brain interregional integration among patients with SCZ, which is consistent with a substantial body of prior evidence.^[^ ^2^, ^35^, ^36^ ^]^ Specifically, the extent of synergy decline corresponded with the cortical information‐processing hierarchy, with primary SOM and VIS subnetworks exhibiting minimal reduction, while higher‐order DMN and FPN subnetworks, which are crucial for complex tasks dependent on information integration, experienced the most significant impairment. This observation indicates that brain integration deficits in SCZ could affect a spectrum of functions along the neurocognitive architecture. Overall, our findings emphasize the importance of synergistic interactions, the disruption of which could lead to a range of functional abnormalities in SCZ.

The second key finding was the successful decomposition of the brain‐wide atypical synergistic interaction pattern into three dissociable factors at the individual level, each demonstrating clinical and biological specificity. Factor 1 primarily entailed a synergy reduction between the SUB and LIM subnetworks and other brain regions; Factor 2 was related to synergy reduction involving the VIS subnetwork and other brain subnetworks; and Factor 3 primarily represented a synergy decrease between the association cortices of the DMN, FPN, and other cortical regions. Notably, our analysis was based on a Bayesian framework using the LDA algorithm,^[^ ^16^, ^18^ ^]^ which was chosen in consideration of the increasingly acknowledged fact that variations in neuroimaging signatures and clinical phenotypes in psychiatry constitute a dimensional continuum.^[^ ^14^, ^37^ ^]^ This method uncovers latent factors, enabling variable expressions among individuals and capturing interindividual differences in factor expression profiles. Moreover, this method may align with the concept that SCZ encompasses the overlap of multiple pathological mechanisms, an aspect that has not been adequately addressed in the category subtype approach.^[^ ^12^, ^15^ ^]^ Our results further revealed that most patients with SCZ expressed more than one factor, further underscoring the necessity of using this decomposition method.

Factor 1 expression loadings were exclusively associated with positive symptoms. This clinical relevance is consistent with multiple lines of evidence. First, the factor 1 pattern includes atypical synergistic interactions between the subcortical and cortical regions, with existing evidence linking the aberration of this circuit to positive symptoms.^[^ ^38^, ^39^ ^]^ For example, corticostriatal functional abnormalities could serve as predictors of positive symptoms in SCZ.^[^ ^39^ ^]^ Second, the factor 1 pattern was predominantly correlated with cognitive functions, such as emotion and pain in the Neurosynth database. These cognitive deficits are closely associated with positive symptoms.^[^ ^40^ ^]^ Third, our results further showed significant correlations between the Factor 1 pattern and molecular imaging of neurotransmitters, including 5‐HT and dopamine receptors. These neurotransmitters, particularly dopamine D2 receptors, are implicated in the pathophysiology of SCZ, and serve as the main targets for antipsychotic medications, while their blockade leads to an improvement in positive symptoms.^[^ ^41^, ^42^ ^]^

Factor 2 expression loadings were unrelated to psychiatric symptoms, but its pattern was associated with specific visual and sensory functions, such as visuospatial and multisensory processing, which could be attributed to the fact that synergy impairment was most prominent in the VIS subnetwork. However, factor 2 showed significant associations with certain SCZ‐implicated neurotransmitter systems, such as 5‐HT1a and Mu‐opioid receptors,^[^ ^43^, ^44^ ^]^ as well as with subcortical input parameter differences in the biophysical model. As such, we hypothesized that factor 2 might exert its influence on clinical aspects in a more indirect manner.

Factor 3 loadings were linked to both negative and general psychological symptoms. Examination of the Factor 3 pattern primarily revealed reduced synergistic interactions within the cortex, particularly among the DMN and FPN networks and other brain regions. Numerous neuroimaging findings have suggested that disruptions in cortical neural activity can lead to negative and general psychopathological symptoms, thereby reinforcing our observations regarding the connection between factor 3 and clinical symptoms.^[^ ^45^, ^46^ ^]^ Further, the factor 3 pattern was significantly related to higher‐level cognitive domains, such as memory and social cognition, which overlapped with general psychopathological symptoms to some extent.^[^ ^47^, ^48^, ^49^ ^]^ Furthermore, the factor 3 pattern was correlated with certain neurotransmitter systems, such as cannabinoids and nicotinic receptors, which have been reported to be related to negative symptoms.^[^ ^50^, ^51^, ^52^ ^]^ Finally, the connection between the recurrent connection parameter difference in the biophysical simulation and the factor 3 pattern supports the atypical synergistic interaction from a microcircuit perspective.

Our analysis integrating neuroimaging and transcriptomics revealed that the gene sets most correlated with the three‐factor patterns were significantly enriched in SCZ, providing genetic validation for SCZ specificity, and supporting the notion that SCZ is a highly heritable psychiatric disorder.^[^ ^53^ ^]^ Additionally, factors 1 and 3 demonstrated significant correlations with gene co‐expression patterns, implying a pivotal role for genetic factors in shaping their patterns and their associations with clinical and neurophysiological phenotypes, indicating the genetic specificity of these three factors.

Our study has several limitations. First, information decomposition is performed at the brain parcellation level. Although we performed control analyses using different brain parcellation schemes, this relatively coarse‐grained approach may have obscured certain locally significant abnormalities. Future studies should focus on voxel‐level analyses, particularly when investigating specific nuclei and brain circuits. Second, the factor decomposition method employed comprised a fully data‐driven framework, which may impose certain requirements on the quality and length of resting‐state fMRI time‐series data, as well as the number and distribution of individuals. Therefore, further validation of the atypical synergistic interaction pattern and the three factors that decompose into various larger datasets is required to ensure generalizability. Third, three latent factors were obtained from resting‐state fMRI data. However, these factors may organize differently when individuals engage in specific tasks. Further exploration should be performed by analyzing task‐based fMRI data. Last, the transcriptome and PET/SPECT datasets were developed predominantly from Caucasian brains, and thus may not entirely reflect the physiology of the Chinese participants in our analysis. Therefore, it is crucial to verify these findings in the future by integrating multi‐omics (genetic, molecular imaging, and fMRI) data from the same sample.

## 4 Conclusion

In summary, our investigation revealed a widespread reduction in synergistic interactions among patients with SCZ, with these aberrant patterns exhibiting considerable heterogeneity that could be further characterized as three distinct latent factor modes. Each patient further expressed different factors to varying degrees, with each factor specifically aligned with the clinical symptom profiles and neurobiological underpinnings. Our findings highlight the crucial role of synergy, while dissection into three SCZ‐specific factors helped unravel the complexity of SCZ and provide new evidence to help understand its pathophysiological mechanisms.

## 5 Experimental Section

### Participants

Primary analysis was conducted using a multisite dataset, for which all enrolled participants were recruited from seven sites at six hospitals: Peking University Sixth Hospital (PKU6), Beijing Huilongguan Hospital (HLG), Xijing Hospital (XIAN), Henan Mental Hospital (XX_S; XX_G), Renmin Hospital of Wuhan University (WUHAN) and Zhumadian Psychiatric Hospital (ZMD). MRI data were collected using a 3T Siemens Scanner (four sites: PKU6, HLG, XIAN, and XX_S) or a 3T GE scanner (three sites: XX_G, WUHAN, and ZMD). The same scanning protocols were used at all sites to avoid confusion caused by different scanning parameters. Two scanners were used at the Henan Mental Hospital; therefore, the participants at Henan Mental Hospital were divided into two sites according to the scanner used. There was no overlap in participants between the two sites. All the participants and/or their legal guardians provided written informed consent to participate. The local medical ethics committee approved the study protocol. All patients with SCZ were diagnosed by two qualified psychiatrists as meeting the SCZ criteria utilizing the Structured Clinical Interview for DSM‐IV axis I disorders (SCID‐I/P, Patient Edition). The PANSS was used to evaluate the psychotic symptoms in patients with SCZ. Only patients who scored >60 on the total score and > 4 on at least three positive items, were included. Normal control individuals (NC) were also recruited from each hospital and screened using the SCID‐I, Non‐Patient Edition (SCID‐I/NP). Detailed eligibility and exclusion criteria for the participants have been described in the previous study.^[^ ^2^ ^]^ Among them, 1078 subjects (538 individuals with SCZ and 540 NC) with high‐quality MRI data were included after a series of quality control measures. Non‐clinical characteristics such as gender, age, and education length, and complete PANSS score information (only for SCZ patients) were also collected. MRI data quality was ensured by the following steps: screening artifacts, inspecting head motion (removing participants with maximum absolute translation >3 mm or maximum absolute degree >3°), and checking the registration and normalization quality in the preprocessing section. Participants’ clinical and non‐clinical characteristics at each site were shown in Table S1 (Supporting Information).

After initial analysis, the COBRE and UCLA datasets were utilized for the control analysis. Briefly, the COBRE dataset containing multimodal MRI data of SCZ and NC was shared by the Mind Research Network and the University of New Mexico, and can be accessed via the website (https://coins.trendscenter.org/).^[^ ^22^, ^54^ ^]^ Written informed consent was obtained from each participant. Patients with SCZ were diagnosed utilizing the Structured Clinical Interview for DSM‐IV. The UCLA dataset containing multimodal MRI data of NC and patients with neuropsychiatric disorders including SCZ, BP, and ADHD, can be openly accessed via a website (https://openneuro.org).^[^ ^55^ ^]^ All participants provided written informed consent after the procedures were approved by the Institutional Review Boards of UCLA and the Los Angeles County Department of Mental Health. Patients with SCZ were diagnosed using the Structured Clinical Interview for DSM‐IV Axis I Disorders (SCID‐I). Following the same quality control measures and preprocessing procedures as the multi‐site dataset, 70/70 SCZ/NC from the COBRE dataset and 47/115 SCZ/NC from the UCLA dataset, were included in the control analysis. Given that on computing the mean and variance of synergistic interactions were focused for the NC group to normalize the SCZ group in the control analysis, it was believed that the disparity in sample sizes between the NC and SCZ groups may not be a critical concern.

### MRI Acquisition

For the multi‐site dataset, resting‐state fMRI, T1w, and DTI data were included in the current study. T1‐weighted images were collected using rapid gradient‐echo sequence with the following parameters: matrix size = 256 × 256 × 192 (Siemens scanners) or 256 × 256 × 188 (GE scanners); voxel size = 1 × 1 × 1mm^3^; inversion time (TI) = 1100 ms; slice thickness = 1 mm. Resting‐state fMRI data was acquired using an echo planar imaging (EPI) sequence with the following parameters: repetition time = 2000 ms; echo time = 30 ms; flip angle (FA) = 90°; matrix size = 64 × 64; voxel size = 3.4375 × 3.4375 × 4.6mm^3^; slice thickness = 4 mm; gap between slices = 0.6 mm; slices = 33; volumes = 240 (PKU6, HLG, XIAN, XX_S, XX_G, WUHAN; 8 min) or 180 (ZMD; 6 min). The DTI data was acquired using a single‐shot spin‐echo EPI (SE‐EPI) sequence from the participants of the PKU6 with the following parameters: matrix size = 128 × 128; voxel size = 2 × 2 × 3 mm^3^ and without slice gap; FOV = 256 × 256 mm^2^; slices = 50; 64 noncollinear diffusion gradient directions; 64 volumes with b‐value = 1000 s mm^−2^ and 1volume with b‐value = 0 s mm^−2^.

For the COBRE dataset, MRI data was acquired by echoplanar imaging from a 3T Siemens TimTrio scanner, with available scanning parameters in the previous study.^[^ ^54^ ^]^ For the UCLA dataset, fMRI data were obtained by an echoplanar imaging sequence from two 3T Siemens Trio scanners, with available scanning parameters in the previous study.^[^ ^55^ ^]^

### MRI Preprocessing

All resting‐state fMRI data from the multi‐site, COBRE, and UCLA datasets, were uniformly preprocessed using BRANT version 3.35,^[^ ^56^ ^]^ a MATLAB toolbox integrating standardized procedures and tools for preprocessing fMRI data. The initial ten volumes were discarded to stabilize the magnetization. The standardized preprocessing procedures comprised the following steps: correction for slice timing (to address temporal disparities among different slices); intra‐participant realignment of EPI images (for the estimation and correction of head movements across an individual's EPI volumes); rigid‐body transformation‐based registration from T1‐weighted (T1w) to the mean EPI image; spatial normalization of EPI images to the standard Montreal Neurological Institute (MNI) space; resampling of the normalized EPI images to a voxel size of 3 × 3 × 3 mm^3^; removal of potential confounding effects by employing a multiple regression model to eliminate linear trends, the influence of white matter (WM) and cerebrospinal fluid (CSF), the first derivatives of WM and CSF, as well as head motion estimates obtained during the realignment step; and temporal band‐pass filtering within the range of 0.01–0.08 Hz to mitigate low‐frequency drifts and physiological noise. The filtered signals were averaged within each brain region to obtain time‐series data for subsequent information decomposition.

The T1w data from the multi‐site dataset were preprocessed using CAT12, an extension toolkit of SPM12, to calculate GMV. Standardized preprocessing procedures were further performed, including denoising, intensity normalization, and linear and nonlinear registration using the MNI152 template. Subsequently, the segmented gray matter images were normalized and resampled to a voxel size of 1.5 × 1.5 × 1.5 mm^3^. The normalized gray matter images were subsequently modulated by multiplying the voxel values with the Jacobian determinant derived from spatial normalization and smoothed using a Gaussian kernel of 8 × 8 × 8 mm^3^ full‐width at half maximum. Finally, the gray matter images were averaged within each brain region to obtain regional GMV for each participant.

The DTI data of participants at the PKU6 site (90 SCZ and 99 NC) was preprocessed to correct for distortions resulting from head motion and eddy currents, and to subsequently construct structural connectivity. The registration tools *flirt* and *fnirt* in FSL were employed to register from DTI space to T1 space and from T1 space to standard space. MRtrix toolbox was employed to estimate the response functions by *dwi2response* tool,^[^ ^57^ ^]^ generate the tractogram with 1 million streamlines, and reconstruct white matter pathways and streamlines tractography. Due to limitations imposed by the neural dynamics model, on cortical regions were focused exclusively, and built the structural connectome by mapping the reconstructed streamlines onto the Schaefer version‐100 atlas.

### Brain Parcellations and Canonical Resting‐State Subnetworks

Human brain regions were delineated into 100 cortical and 15 subcortical regions, collectively referred to as the “Schaefer‐115′ parcellation. The 100 cortical regions of interest (ROIs) originated from the 100 functional parcellations, initially developed from cortical intrinsic functional connectivity by Schaefer et al.^[^ ^58^ ^]^ Given that this parcellation exclusively covers cortical regions, 15 subcortical regions were further included (thalamus, caudate, putamen, pallidum, accumbens, hippocampus, amygdala in both left and right hemispheres, and ventricles) from the Desikan–Killiany parcellation to construct the whole‐brain atlas.^[^ ^25^ ^]^ In the context of canonical resting‐state subnetworks, the established canonical cortical parcellation was employed, encompassing seven subnetworks initially developed by Yeo et al.^[^ ^21^ ^]^ These subnetworks were categorized as visual (VIS), somatomotor (SOM), salience/ventral attention (SAL), dorsal attention (DAN), limbic (LIM) frontoparietal (FPN), and default mode (DMN). Each of the 100 cortical ROIs from the functional parcellation belongs to one of these resting‐state canonical subnetworks, according to the developer. Thus, all the 15 subcortical regions was incorporated into a subcortical subnetwork termed ‘SUB.”

### Functional Connectivity

Pearson's correlation was calculated between all pairs of regional fMRI time‐series, yielding a 115 × 115 FC matrix for each participant. Confounders including age, gender, head motion, and site, were regressed out from each FC entry using a general linear model.

### Synergy and Redundancy Inferred by Integrated Information Decomposition

To calculate the synergistic and redundant interactions among brain regions, a recently introduced Integrated Information Decomposition method was employed,^[^ ^7^ ^]^ which enables a more intricate examination of the information dynamics underlying the fMRI time‐series.^[^ ^7^, ^8^, ^59^ ^]^ Herein, a concise overview of its underlying concepts were provided, with comprehensive details available in the original methodology section. The concept of Partial Information Decomposition (PID) represents an extension of Shannon's mutual information theory, demonstrating that the mutual information *I*(*X*, *Y*; *Z*) offered by multiple sources (Taking two sources as an example: *X*, *Y*) to target *Z* can be decomposed into three partial information atoms (Equation (1)): unique information (information exclusively offered by only one source), redundant information (information offered individually by both sources), and synergistic information (information jointly offered by their combined influence):^[^ ^60^ ^]^

(1) $$\begin{array}{c} I\;\left(X,Y;Z\right)=\;\mathrm{red}\left(X,Y;Z\right)+\mathrm{uni}\left(X;Z\left|Y\right.\right)+\mathrm{uni}\left(Y;Z\left|X\right.\right)+\mathrm{syn}\left(X,Y;Z\right) \end{array}$$

Integrated Information Decomposition extends this concept to dynamic systems.^[^ ^7^ ^]^ Taking system *X*, characterized by two time‐series containing information on the intrinsic interaction: $X_{t}=\;\left(X_{t}^{1};X_{t}^{2}\right)$, as an example. The PID method considers the $X_{t}^{1}$ and $X_{t}^{2}$ as sources and *X_t_*_+1_ as the target to obtain four PID atoms (as shown in Equation (1): Syn, Uni,^1^ Uni,^2^ and Red). The pivotal advancement introduced by Integrated Information Decomposition lies in its ability to provide a comprehensive decomposition of information, encompassing 4×4 ΦID atoms (pairs of PID atoms, for example, Red→Red: the redundant information in the past continues to be redundant in the future; Red→Uni^1^: the redundant information in the past transforms into unique information of $X_{t+1}^{1}$ in the future; and so forth) across multiple sources and multiple targets simultaneously, by considering the time‐reverse of PID (“backwards” PID). Common information‐theoretic measures such as transfer entropy and causal density, can further be expressed as diverse combinations of the 16 ΦID atoms. In the case, the time‐series from each pair of brain regions were considered as system *X*, and computed 16 ΦID using minimum mutual information, as defined in Equation (2):

(2) $$\mathrm{red}\;\left(X_{t-\tau };X_{t}\right)=\;\mathrm{mi}n_{ij}I\left(X_{t-\tau }^{i};X_{t}^{j}\right)$$

Following the previous approach, the stable synergy and redundancy were only considered over the time scale, e.g., Red→Red and Syn→Syn of the 16 ΦID atoms.

### Latent Factors Inferred by Bayesian Model

The hierarchical Bayesian Latent Dirichlet allocation (LDA) model was originally created to autonomously unveil hidden themes within a compilation of articles.^[^ ^16^, ^18^, ^61^, ^62^ ^]^ This model operates under the assumption that each article constitutes an unordered assemblage of words correlated with specific hidden themes. Each theme was characterized by the expected frequency of each word. The likelihood of a particular word in the lexicon for each theme, and the likelihood that each theme was linked to a specific article can further be obtained simultaneously using specialized algorithms.^[^ ^16^ ^]^ In the study, the LDA model was adapted to the SCZ synergy matrices. Individuals with SCZ were likened to the articles, latent synergy factors were equated to hidden themes, and synergistic interactions between brain regions served as the words in the lexicon.

To elaborate, the process was initiated by standardizing the synergy matrices within each individual with SCZ, and aligning them with the corresponding distribution in the control group through *z*‐score normalization. Subsequently, each synergy matrix assumed the role of an “article,” with positive *z*‐scores reset to zero, and absolute values taken for negative *z*‐scores, given that the SCZ predominantly manifested as a reduction in synergy. Concatenating these preprocessed feature vectors across subjects culminated in a matrix featuring 538 rows (each representing an individual) and 6555 columns (comprising vectorized synergy features). Finally, the LDA model was employed on the preprocessed synergy dysfunction patterns of participants with SCZ to derive estimations of the latent factors. By employing a hyper‐parameter that specifies the number of synergy factors, the likelihood can ascertain that each participant expresses each synergy factor (namely factor expression loading), along with the factor‐specific dysfunction patterns.

### Neuroimaging–Clinic Association Analysis

An exploration was embarked on to investigate whether and how the composition of LDA‐decomposed synergy factors correlated with individual variations in nonclinical and clinical characteristics among participants with SCZ. To achieve this, an approach inspired was employed by representational similarity analysis (RSA) to probe the individual variabilities of these latent factors.^[^ ^26^, ^63^ ^]^ Subject dissimilarity was first computed based on three demographic variables (gender, scanning site, and age) and four PANSS scores (total, positive, negative, general). For numerical variables such as age and PANSS score, subject dissimilarity was quantified as the absolute Euclidean distance between measurements, reflecting the difference in age or PANSS scores. For categorical variables such as gender or scanning site, subject dissimilarity was set to 0 if the categorical variables matched, and 1 if they did not. Following this, subject dissimilarity with respect was calculated to the original synergy *z*‐score matrices and estimated the three‐factor expressions. To ensure the robustness of the RSA analyses across different random initializations of the LDA, the latent factor expressions were sampled from 100 random runs. Unpaired sample *t*‐tests were subsequently employed to determine whether the representational dissimilarity matrices (RDMs) for participants’ demographic properties and symptoms exhibited stronger correlations (Kendall rank correlation *τ*) with the RDMs based on the individual's original synergy pattern or with the RDMs based on the LDA‐decomposed synergy factor expressions.

Moving beyond the exploration of associations between participants’ characteristics and overall synergy factor expressions through RSA, the relationships were examined between each synergy factor and participants’ characteristics. Initially, Pearson correlations (FDR‐corrected) were computed between the three factors and the three total scores of the positive, negative, and general PANSS subscales, with non‐clinical characteristics regressed out from PANSS scores and factor expressions. The correlations were further explored between each factor and each of the 30 PANSS item scores.

Last, to explore and validate how the expression of the synergy factors contributed to non‐clinical characteristics, a GLM was employed to compare age, gender, head motion, and education length across the three synergy factors. The site effect was implied by the factor composition; therefore, the site was regressed out of the characteristics of interest and factor expressions before GLM. Thus, the GLM was formulated as *Y* = 𝛽_0_ + 𝛽_1_𝑝_1_ + 𝛽_2_𝑝_2_ + 𝜖, where 𝑝_1_ (𝑝_2_) represented factor 1 (2) expressions of all participants, **𝛽** denoted the corresponding regression coefficients, and *Y* indicated the target characteristics. The expression of factor 3 (𝑝_3_) was implicitly modeled because 𝑝_1_ + 𝑝_2_ + 𝑝_3_ = 1, and 𝛽_0_ can be considered as the regression coefficient of 𝑝_1_ + 𝑝_2_ + 𝑝_3_. Statistical tests were further conducted to examine variations in the characteristic *Y* across the three synergy factors. Null hypotheses in these tests were expressed as **H*β*** = 0, where ***β*** = [*β* ^0^, *β*,^1^ *β* ^2^]^𝑇^, and **H** = [0, –1, 1] for comparing factor 1 and 2, or **H** = [0, 1, 0] for comparing factor 1 and 3, or **H** = [0, 0, 1] for comparing factor 2 and 3.

### Neuroimaging–Transcription Association Analysis

To provide further neurobiological underpinnings of each factor, the potential mechanisms were explored underlying the synergy factors using the Allen Human Brain Atlas transcriptome dataset.^[^ ^19^ ^]^ This dataset comprises regional microarray expression data from the postmortem brains of six donors (five men and one woman, ages ranging from 24 to 57 years). The Abagen toolbox was further employed to preprocess the gene expression data and map them to the augmented “Schaefer‐115′” parcellation in MNI space.^[^ ^64^ ^]^ Following Abagen's recommended protocol, the preprocessing pipeline consisted of probe reannotation, data filtering, probe selection, sample selection, assignment to brain regions, normalization of gene expression values, and averaging across regions. Specifically, the microarray probes were first re‐annotated using data provided by Arnatkeviciute et al.,^[^ ^65^ ^]^ and subsequently discarded probes that did not match a valid Entrez ID. Second, probes were removed with expression intensities lower than the background intensity in more than half of the samples across donors. Third, when multiple probes indexed the expression of the same gene, the probe with the most consistent regional variation pattern across the donors was selected. Next, the samples were assigned to the augmented ‘Schaefer‐115′ parcellation, if their MNI coordinates were within 2 mm of the given region. The samples that were not assigned to any region were discarded. To reduce the potential for misassignment, sample‐to‐region matching was constrained by hemispheric and gross structural divisions (i.e., cortical and subcortical regions). Gene expression values were normalized across samples within gross structural divisions using a robust sigmoid function and a unit interval rescale operation. Finally, by averaging the expression values of samples assigned to the same region separately for each donor and across donors, this workflow resulted in a regional gene expression matrix with 115 rows (representing brain regions) and 15633 columns (representing the remaining genes).

The matrix‐level synergy dysfunction patterns were subsequently aggregated into vectors for each factor by summing them at the regional level. Pearson's correlations were calculated between regional expression values and factor vectors for each gene. The top and bottom 1500 (≈10%) ranked genes for each factor were selected as the synergy risk gene sets. To assess whether these genes had SCZ specificity, disease enrichment analysis was performed on the positively and negatively correlated gene sets using the ToppGene portal.^[^ ^66^ ^]^ The gene sets were aggregated (*n* = 6913) for the three factors to build a gene co‐expression matrix to investigate the impact of gene co‐expression on these factors. This entailed the calculation of a 115×115 correlation matrix between brain regions using Spearman's correlations for all synergy risk genes, followed by Fisher's *z*‐transformation to create a gene co‐expression matrix. Subsequently, the relationship was calculated, referred to as the gene‐factor correlation, by applying Pearson's correlation to understand the link between regional gene co‐expression and dysfunction patterns of synergy factors. The significance of gene–factor relationships was further assessed using a Spatial Autocorrelation (SA) permutation test.^[^ ^32^ ^]^

Furthermore, to gain deeper insight into the influence of gene co‐expression on synergy factors, a “Virtual Gene Knockout” was applied approach to assess the contribution of each gene to the gene–factor correlation.^[^ ^67^ ^]^ In this method, the removal of each of the 6913 synergy‐specific genes were individually simulated to mimic a gene‐KO scenario. Gene co‐expression networks were further reconstructed without this gene, and evaluated the changes in correlation coefficients between gene co‐expression and factor patterns before and after deletion, which served as the gene contribution indicator (GCI). Based on the GCI values, the 6913 genes were categorized into two sets for each factor: a positive‐contribution gene set (GCI^+^) and a contribution‐negative gene set (GCI^−^). These sets helped to understand the biological roles and impacts of genes on synergy factors. To further elucidate their roles, a gene ontology (GO) was performed enrichment analysis using the ToppGene portal.^[^ ^66^ ^]^ To account for multiple comparisons, the FDR‐BH correction was applied (*p* < 0.05) during the analysis.

### NeuroSynth Term‐Based Meta‐Analysis

To decode the patterns of synergy dysfunction for each factor, the Neurosynth database was utilized (https://neurosynth.org/), an online platform for large‐scale meta‐analyses of fMRI studies, to analyze synergy factor maps. These synergy factor maps were the same as those used in the transcriptional association analysis by aggregating the matrix‐level synergy dysfunction patterns into vectors for each factor at the regional level. Twenty four topic terms were selected that encompassed a fairly comprehensive range of domains of behavior and cognition, as utilized in previous studies.^[^ ^29^, ^30^ ^]^ Each factor map was ranked and divided into 20 sections based on the magnitude of the activation values. For each section, a new brain map was created by setting the values of the remaining regions to zero. The top four brain maps were selected representing the top 20% activation for each factor as inputs for the meta‐analysis. Considering that the topic terms related to factor 2 differed significantly from those related to factors 1 and 3, the 12 brain maps were arranged in the order of factors 2, 1, and 3. Subsequently, the *z*‐scores of the topic terms were weighted using this sequence of values, reordered the topics, and visualized the results on a graph. Visualization excluded the term “cognition” because it did not reach the significance threshold of *z* > 3.1 in any of the 12 brain maps.

### Microscale Neural Dynamic Modeling

A recently developed neural dynamic model was harnessed (parametric mean‐field model, pMFM) to simulate functional dynamic signals and infer microcircuit parameters of the cortical regions.^[^ ^20^ ^]^ The preprocessed DTI and fMRI data from the PKU6 site were used to calculate structural and functional connectivity, which served as input for pMFM. Briefly, the mean‐field model (MFM) was developed by simplifying the spiking neural mass model (NMM) using mean‐field approximations.^[^ ^68^ ^]^ The pMFM builds upon this foundation by parameterizing regional microcircuit parameters with the T1w/T2w ratio and principal FC gradient, and captures both static functional connectivity (FC) and time‐varying functional connectivity dynamics (dFC) more realistically while maintaining the parametric complexity within reasonable limits. In pMFM, the time‐varying functional brain dynamics of each region were described by the following coupled nonlinear stochastic differential equations ((Equations (3),–5)):

(3) $${\dot{S}}_{i}=-\frac{S_{i}}{\tau_{s}}+r\left(1-S_{i}\right)H\left(x_{i}\right)+\sigma v_{i}\left(t\right)$$

(4) $$H\;\left(x_{i}\right)=\frac{ax_{i}-b}{1-\exp(-d\left(ax_{i}-b\right))}$$

(5) $$\;x_{i}=W_{i}\;JS_{i}+GJ\sum_{j}C_{ij}S_{j}+I_{i}$$

The state parameters *S_i_*, *H*(*x_i_*), and *x_i_* denote the mean synaptic gating variable, population firing rate, and total input of the *i*‐th brain region from all the cortical and subcortical regions, respectively. Further details on the descriptions and sets of other parameters were provided in the original study.^[^ ^20^ ^]^ System parameters *W_i_* (recurrent connections), *I_i_* (subcortical inputs), and *σ* (noise) were parameterized in pMFM as linear combinations of principal FC gradient,^[^ ^29^ ^]^ and group‐level T1w/T2w myelin maps ((Equations (6)–(8)):^[^ ^69^ ^]^

(6) $$\;W_{i}=a_{w}\;\mathrm{My}e_{i}+b_{w}\mathrm{Gra}d_{i}+c_{w}$$

(7) $$\;I_{i}=a_{I}\;\mathrm{My}e_{i}+b_{I}\mathrm{Gra}d_{i}+c_{I}$$

(8) $$\;\sigma_{i}=a_{\sigma }\;\mathrm{My}e_{i}+b_{\sigma }\mathrm{Gra}d_{i}+c_{\sigma }$$

According to the original study, the CMA‐ES optimization algorithm was employed to determine optimal system parameters (***W***, ***I***, and *σ*) by minimizing an overall loss of both static FC and dynamic FC (dFC) in the simulations. Loss of static FC was defined as one minus the Pearson's correlation between the upper triangular entries of the simulated and empirical FC matrices. Loss of dFC was defined as the Kolmogorov‐Smirnov distance between the upper triangular entries of the simulated and empirical dFC matrices. The optimization procedure was ran with a five‐fold cross‐validation to validate the application of this model to the data. Considering that mean‐field models emphasize the fitting of FC, Pearson's correlations were compared between the original and simulated FC with Pearson's correlations between the original FC and SC in the training and test sets. Finally, Pearson's correlations were calculated between the between‐group differences (SCZ−NC) of the parameters of interest (recurrent connections ***W***, subcortical inputs ***I***) and the three factor maps to evaluate whether imbalances in these microcircuit parameters correlated to synergy factors. Significances of spatial correlations were assessed using the SA permutation test.

### Spatial Correlation Analysis with PET and SPECT Data

To quantitatively assess molecular and functional enrichment of the three factors, an open toolbox (Neuromaps) was used which collects brain maps capturing molecular, microstructural, electrophysiological, developmental, functional, genetic, neurophysiological, and metabolic features from the previous studies.^[^ ^31^ ^]^ Among them, 36 neurotransmitter receptor or transporter maps obtained from open PET and SPECT data, including serotonin, dopamine, acetylcholine, norepinephrine, glutamate, GABA, synaptic vesicle glycoprotein, cannabinoid, and mu‐opioid, were map‐to‐map compared with the SCZ synergy factor maps. PET data provides information on the spatial distribution of neurotransmitter receptors or transporter profiles, which was valuable for uncovering the molecular substrates associated with these factors. Neuromaps provide a comprehensive set of processing steps and tools, including downloading the target map, transforming it into four standardized coordinate systems, correlating source‐target pairs, and conducting spatial permutations to assess significance. However, for the sake of consistency in this paper, the “SA permutation test” was employed for the significance analysis. Additionally, 17 classic maps were integrated that offered detailed insights into various aspects of neuroanatomy and function to provide context for brain maps related to these three factors.

### Control Analyses

To ensure the robustness and validity of the findings, a series of control analyses were conducted. First, the robustness of the synergy dysfunction pattern was validated using two different parcellations (DK‐83 and BN‐246; Figure S3, Supporting Information). Next, to select the optimal number of factors for LDA inference, the results were compared for two to four latent factors (Figure S5, Supporting Information). To ensure robustness across datasets, all participants were divided into two groups based on MRI scans, and inferred synergy factors independently for each group (Tables S2 and S3; Figure S8, Supporting Information). To ensure the robustness of neuroimaging–clinical association analysis, the Pearson's correlations were computed between the factor expressions and the total scores of the three PANSS subscales for the COBRE dataset (Figure 3c). Next, the associations were compared between the SCZ synergy factors and clinical symptoms by regressing out different control variables (Figure S7b, Supporting Information). The estimates were validated for the COBRE and UCLA datasets, despite their small sample sizes (Table S4, Supporting Information).

### Statistical Analysis

To comprehensively evaluate the spatial correlations of “Schaefer‐115′ maps, Brain Surrogate Maps were employed were employed with Autocorrelated Spatial Heterogeneity (BrainSMASH).^[^ ^32^ ^]^ Specifically, 1000 surrogate maps were generated while rigorously preserving Spatial Autocorrelation (SA) for brain maps to perform permutation test, termed ‘SA permutation test.” The resulting *p*‐value was termed “*p*_SA_.” Unpaired two‐sided *t*‐tests were performed to test the between‐group differences in synergistic and redundant interactions (Figure 1a, Figure S3a,d, Supporting Information), subject mean synergy (Figure 1b, Figure S3c,f, Supporting Information), subject mean redundancy, and FC (Figure S1, Supporting Information), with age, gender, and site controlled as confounders. Unpaired two‐sided *t*‐tests were performed to test the between‐group differences in the regional mean *t*‐statistic values for the three types of interactions (synergy, redundancy, FC, Figure 1c), regional mean synergy (Figure S2c, Supporting Information), PANSS scores (Figure S9, Supporting Information). Unpaired two‐sided *t*‐tests with FDR adjusted were performed to test between‐group differences in model fit of representational similarity analysis (Figure 3b), variance of synergistic interactions for SCZ and NC groups (Figure S4, Supporting Information). Pearson's correlations with SA permutation test were performed to test spatial correlations between the synergy *t*‐statistic map of multi‐site dataset and UCLA/COBRE datasets (Figure 1e), between microcircuit parameters and the spatial patterns of the factors (Figure 4b). Pearson's correlations with FDR adjusted were performed to test correlations between the three factor expressions and three PANSS subscale scores (Figure 3c), 30 PANSS item scores (Figures S7, S8, Supporting Information), CPZ‐eq (Figure S10a, Supporting Information), illness duration (Figure S11a, Supporting Information), and GMV (Figure S12a, Supporting Information). Pearson's correlations with both SA permutation test and FDR adjusted were performed to test correlations between the spatial patterns of the factors and brain maps from Neuromaps (Figures S16, S17, Supporting Information), and the gene expression data (Figure S13, Supporting Information). Pearson's correlations were performed to test correlation between FC and SC in the biophysical computational simulations (Figure S15a, Supporting Information). Kendall rank correlations (Kendall *τ*) were performed to test correlations between the factor expressions (and original synergy *z*‐scores as a control analysis) and the clinical (and non‐clinical) characteristics (Figure 3b). In all cases, significance was defined as *p* < 0.05.

## Conflict of Interest

The authors declare no conflict of interest.

## Supporting information

Supporting Information

## Data Availability

The data that support the findings of this study are available from the corresponding author upon reasonable request.
