# Supplementary material for: Mapping Brain Synergy Dysfunction in Schizophrenia: Understanding Individual Differences and Underlying Molecular Mechanisms
Source: Adv Sci (Weinh). 2024 Jun 20;11(32):2400929. doi: 10.1002/advs.202400929 (PMC11348140; doi:10.1002/advs.202400929)
Supplement: Supplementary file 1 — Supporting Information [file ADVS-11-2400929-s001.pdf]

ADVANCED SCIENCE
Open Access

Supporting Information

for *Adv. Sci.*, DOI 10.1002/advs.202400929

Mapping Brain Synergy Dysfunction in Schizophrenia: Understanding Individual Differences and Underlying Molecular Mechanisms

Chaoyue Ding, Ang Li, Sangma Xie, Xiaohan Tian, Kunchi Li, Lingzhong Fan, Hao Yan, Jun Chen, Yunchun Chen, Huaning Wang, Hua Guo, Yongfeng Yang, Luxian Lv, Huiling Wang, Hongxing Zhang, Lin Lu, Dai Zhang, Zhanjun Zhang, Meng Wang, Tianzi Jiang* and Bing Liu**

Supporting Information

Mapping Brain Synergy Dysfunction in Schizophrenia: Understanding Individual Differences and Underlying Molecular Mechanism

Chaoyue Ding, Ang Li, Sangma Xie, Xiaohan Tian, Kunchi Li, Lingzhong Fan, Hao Yan, Jun Chen, Yunchun Chen, Huaning Wang, Hua Guo, Yongfeng Yang, Luxian Lv, Huiling Wang, Hongxing Zhang, Lin Lu, Dai Zhang, Zhanjun Zhang, Meng Wang, Tianzi Jiang,* Bing Liu**

Table of Contents

Supplementary Table 3

Table S1. Demographic and clinical characteristics of schizophrenia participants, stratified according to site. 3

Table S2. Demographic and clinical characteristics of schizophrenia patients in half-split control analysis. 4

Table S3. Correlations across factors in split-half control analysis 5

Table S4. Control analysis for independent datasets. 5

Supplementary Figure 6

Figure S1. Subject mean redundancy and FC showed no significant between-group differences at each site. 6

Figure S2. Comparison of synergy between SCZ and NC at the levels of connection matrices, brain regions, and functional networks. 7

Figure S3. Synergy networks dysfunction in schizophrenia are robust to the use of brain parcellations 8

Figure S4. Synergistic interactions showed significantly higher inter-individual variance among SCZ compared to NC ... 9

Figure S5. Correlations between the final estimate and solutions from the 100 random initializations in two-, three-, and four-factor estimates. 10

Figure S6. Differences in age, sex, education length and head motion across factors. 11

Figure S7. Associations between synergy factors and symptoms. 12

Figure S8. Associations between SCZ synergy factors and clinic symptoms in split-half control analysis. 13

Figure S9. Three SCZ subgroups stratified based on factor compositions and between-group differences in PANSS scores. 14

Figure S10. The relationship between the SCZ synergy factors and antipsychotic medication at scanning. 15

Figure S11. The relationship between the SCZ synergy factors and illness duration. 16

Figure S12. The relationship between the SCZ synergy factors and total gray matter volume. 17

Figure S13. Transcriptomic correlates of synergistic interaction dysfunction patterns in three schizophrenia latent factors. 18

Figure S14. Validation analysis for three schizophrenia-specific factors from gene perspective. 19

Figure S15. Microcircuit parameters and biophysical simulations. 20

Figure S16. Application of Neuromaps to contextualize three synergy factor maps. 21

Figure S17. Use of Neuromaps to contextualize brain maps of the three factors 22

Reference 23

Supplementary Table

Table S1. Demographic and clinical characteristics of schizophrenia participants, stratified according to site.

sites	PKU6	HLG	XIAN	XX_S	XX_G	WUHAN	ZMD
**Number (SCZ/NC)**	90/98	81/59	83/54	77/102	47/69	78/89	82/69
**Age (y) (SCZ/NC)**	27.4 (6.7)/ 25.8 (5.3)	29.6 (8.7)/ 25.3 (5.4)	25.7 (6.4)/ 31.0 (6.9)	26.0 (5.3)/ 29.2 (7.2)	29.5 (7.5)/ 30.8 (7.2)	24.7 (4.7)/ 26.2 (6.3)	29.8 (7.5)/ 34.2 (7.5)
**Gender (M/F) (SCZ/NC)**	56/34 53/45	32/49 31/28	46/37 31/23	40/37 53/49	28/19 35/34	28/50 46/43	48/34 28/41
**Duration (y) (SCZ)**	4.7 (4.6)	5.9 (5.5)	1.8 (2.4)	3.2 (3.2)	3.9 (4.8)	4.0 (3.9)	4.7 (4.6)
**PANSS positive (SCZ)**	23.7 (4.5)	26.2 (3.1)	23.0 (4.9)	24.5 (3.8)	22.8 (2.6)	23.8 (3.8)	24.9 (4.5)
**PANSS negative (SCZ)**	18.2 (5.9)	16.5 (3.2)	22.7 (6.6)	23.9 (6.0)	19.5 (5.3)	21.2 (5.8)	20.6 (5.8)
**PANSS general (SCZ)**	35.6 (5.4)	36.0 (4.0)	45.0 (8.5)	39.9 (6.2)	39.4 (5.4)	43.7 (7.5)	40.2 (6.5)
**PANSS total (SCZ)**	77.5 (9.9)	78.7 (7.1)	90.8 (15.3)	88.4 (11.6)	81.8 (8.3)	88.7 (12.1)	85.6 (13.0)
**MR scanner (SCZ/NC)**	Siemens Trio 3T	Siemens Trio 3T	Siemens Trio 3T	Siemens Verio 3T	GE Signa HDx 3T	GE Signa HDxt 3T	GE Signa HDxt 3T
**Mean FD (mm) (SZ/NC)**	0.14 (0.07)/ 0.11 (0.04)	0.16 (0.11)/ 0.15 (0.08)	0.13 (0.07)/ 0.13 (0.07)	0.13 (0.08)/ 0.12 (0.06)	0.14 (0.07)/ 0.14 (0.08)	0.12 (0.10)/ 0.10 (0.05)	0.14 (0.09)/ 0.13 (0.09)

Table S2. Demographic and clinical characteristics of schizophrenia patients in half-split control analysis.

	SCZ_S	SCZ_G
**Number (SCZ/NC)**	331/313	207/227
**Age (y) (SCZ/NC)**	27.4 (6.7)/25.8 (5.3)	29.5 (7.5)/30.8 (7.2)
**Gender (M/F) (SCZ/NC)**	174/157<br>168/145	104/103<br>109/118
**Duration (y) (SCZ)**	3.9 (4.6)	4.2 (4.8)
**PANSS positive (SCZ)**	24.2 (5.8)	24.1 (4.5)
**PANSS negative (SCZ)**	19.3 (5.9)	21.5 (6.2)
**PANSS general (SCZ)**	37.8 (7.8)	43.1 (7.8)
**PANSS total (SCZ)**	81.3 (10.3)	88.7 (13.7)
**MR scanner (SCZ/NC)**	Siemens	GE
**Mean FD (mm) (SCZ/NC)**	0.14 (0.07)/0.11 (0.04)	0.13 (0.07)/0.12 (0.08)

Table S3. Correlations across factors in split-half control analysis. a) Correlations between latent factors in the two half-split samples and factors inferred from the whole sample. b) Correlations between factors in the two half-split samples.

a

**Original factors**	**Factor 1**	**Factor 2**	**Factor 3**
**SCZ_S factors**	0.92	0.89	0.86
**SCZ_G factors**	0.88	0.85	0.85

b

	**SCZ_G factor 1**	**SCZ_G factor 2**	**SCZ_G factor 3**
**SCZ_S factors**	0.78	0.72	0.73

Table S4. Control analysis for independent datasets. Correlations between original factors and estimated factors for COBRE[1] and UCLA[2] datasets.

**Original factors**	**Factor 1**	**Factor 2**	**Factor 3**
**COBRE factors**	0.75	0.43	0.38
**UCLA factors**	0.78	0.47	0.42

Supplementary Figure

a

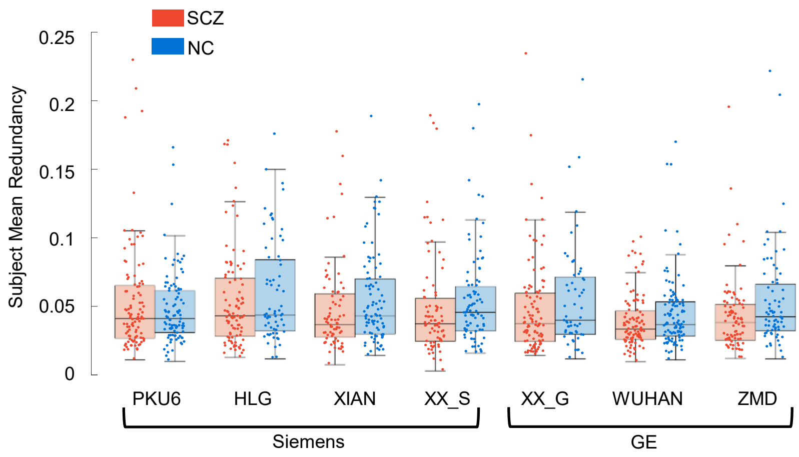

b

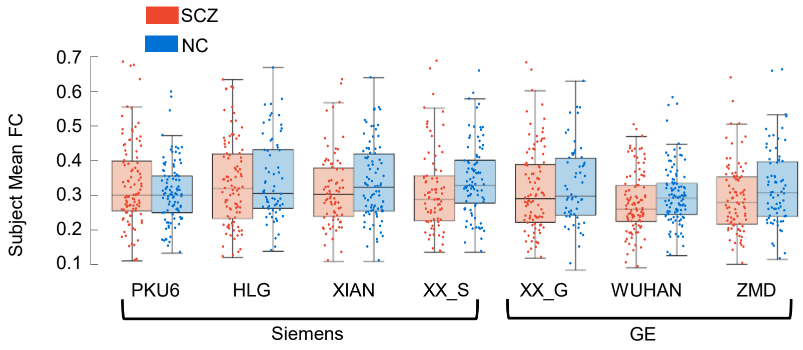

Figure S1. Subject mean redundancy and FC showed no significant between-group differences at each site. a) Scatter-box chart of the mean redundancy of schizophrenia groups (SCZ, red) and normal controls (NC, blue) across different sites. b) Scatter-box chart of the mean redundancy of schizophrenia groups (SCZ, red) and normal controls (NC, blue) across different sites. Each colored circle represents one subject. In the box plots, horizontal lines indicate the median and the hinges of the box denote the first and third quartiles above and below. The lower and upper whiskers represent 1.5 times the interquartile range (IQR).

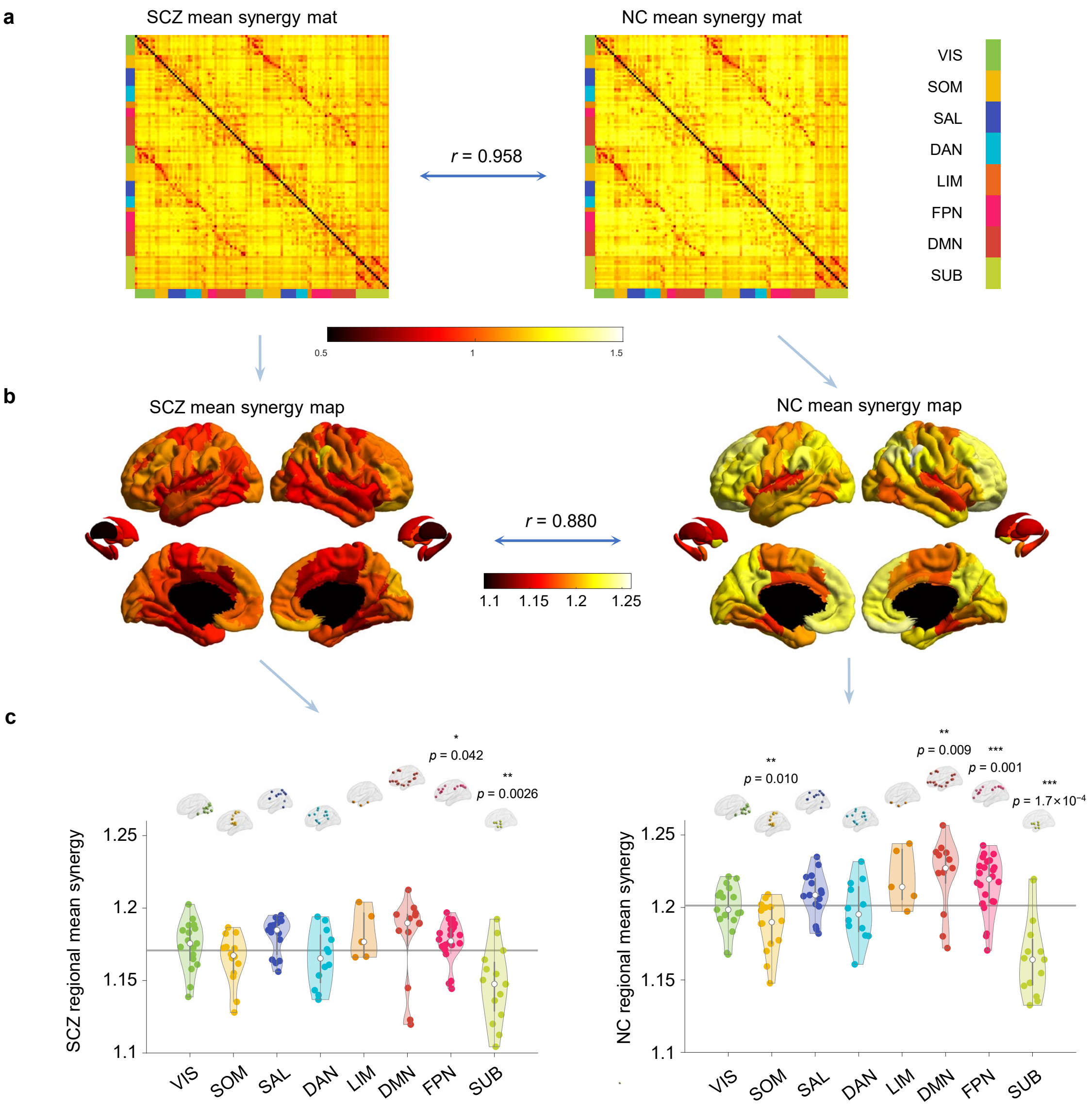

Figure S2. Comparison of synergy between SCZ and NC at the levels of connection matrices, brain regions, and functional networks. a) Group-average synergy matrices display the synergistic interactions between each pair of brain regions (left: SCZ, right: NC). The SCZ synergy showed a similar (Pearson's correlation: r = 0.958) but overall decreased connectivity pattern compared to the NC group. b) Brain plots show regional mean synergy maps calculated by averaging the synergy matrices across brain regions for SCZ and NC groups. The SCZ synergy showed a similar (Pearson's correlation: r = 0.88) but overall decreased regional pattern compared to the NC group. c) Violin plot shows the distribution of brain regions assigned to the functional subnetwork[3] indicated on the x axis. White circles represent median values; box limits represent upper and lower quartiles and whiskers represent 1.5× the interquartile range. Significance levels are denoted by asterisks (* indicating $p < 0.05$, ** indicating $p < 0.01$, *** indicating $p < 0.01$), determined by one-sample non-parametric permutation t-test (two-sided) compared to the mean value of whole brain (gray line), without correction for multiple comparisons. In the NC group (right), the synergistic connectivity of the SOM network (primary motor cortex; modular dissociative network) was significantly lower than the overall brain average, while the FPN and DMN (higher-order association networks; modular integrated networks) exhibited significantly higher synergy levels, consistent with the definition of synergy where two brain regions depend on each other to generate new information. However, in the SCZ group (left), this network-specific differentiation lost significance, particularly in the SOM and DMN networks, underscoring the importance of examining synergistic factors from a whole-brain perspective.

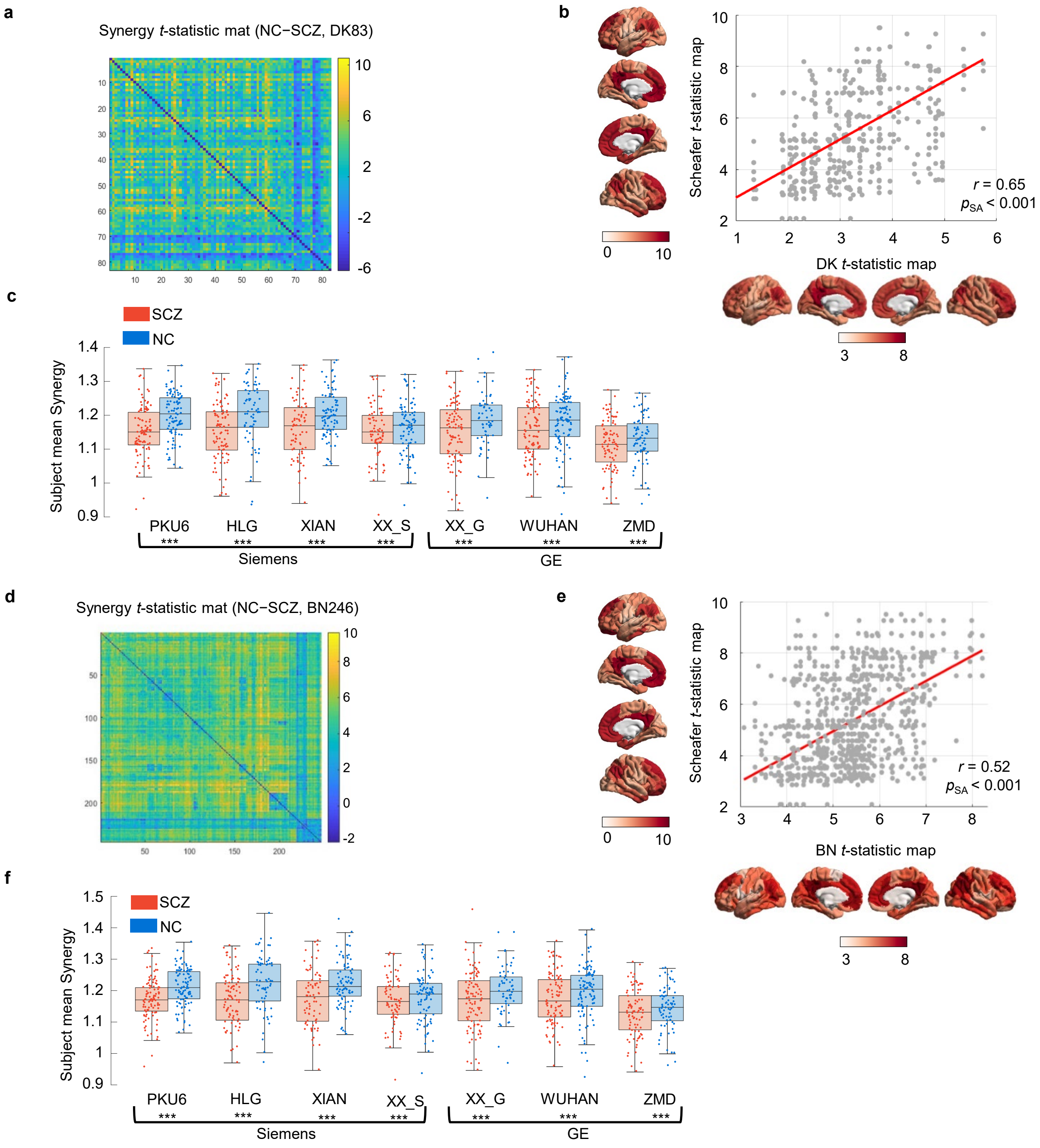

Figure S3. Synergy networks dysfunction in schizophrenia are robust to the use of brain parcellations. a) Matrix of *t*-statistic values display between-group differences in synergistic interactions within the DK-83 parcellation,[4] derived from Desikan-Killiany anatomical atlas encompassing 68 cortical regions and 15 subcortical regions. b) Robustness of synergy *t*-statistic map to DK-83 parcellation. Significant correlation between augmented Scheafer-115 parcellation and DK-83 parcellation ($r = 0.65$, $p_{SA} < 0.001$) underscores the resilience of synergy dysfunction to various parcellation schemes. c) Scatter-box chart of the mean synergy of schizophrenia groups (SCZ, red) and normal controls (NC, blue) across different sites using DK-83 parcellation. Synergy networks dysfunction in the schizophrenia group was evident at all independent sites, despite variations in scanning equipment. d) Matrix of *t*-statistic values display between-group differences of the synergistic interactions in the BN-246 parcellation,[5] obtained by brainnetome anatomical atlas with 210 cortical regions and 36 subcortical regions. e) Robustness of synergy *t*-statistic map to BN-246 parcellation. Significant correlation between augmented Scheafer-115 parcellation and BN-246 parcellation ($r = 0.65$, $p_{SA} < 0.001$) confirms the robustness of synergy dysfunction to different parcellation schemes. f) Scatter-box chart of the mean synergy of schizophrenia groups (SZ, red) and normal controls (NC, blue) across different sites using DK83 parcellation. Synergy networks dysfunction in the schizophrenia group was evident at all independent sites, despite acquisition using different scanners.

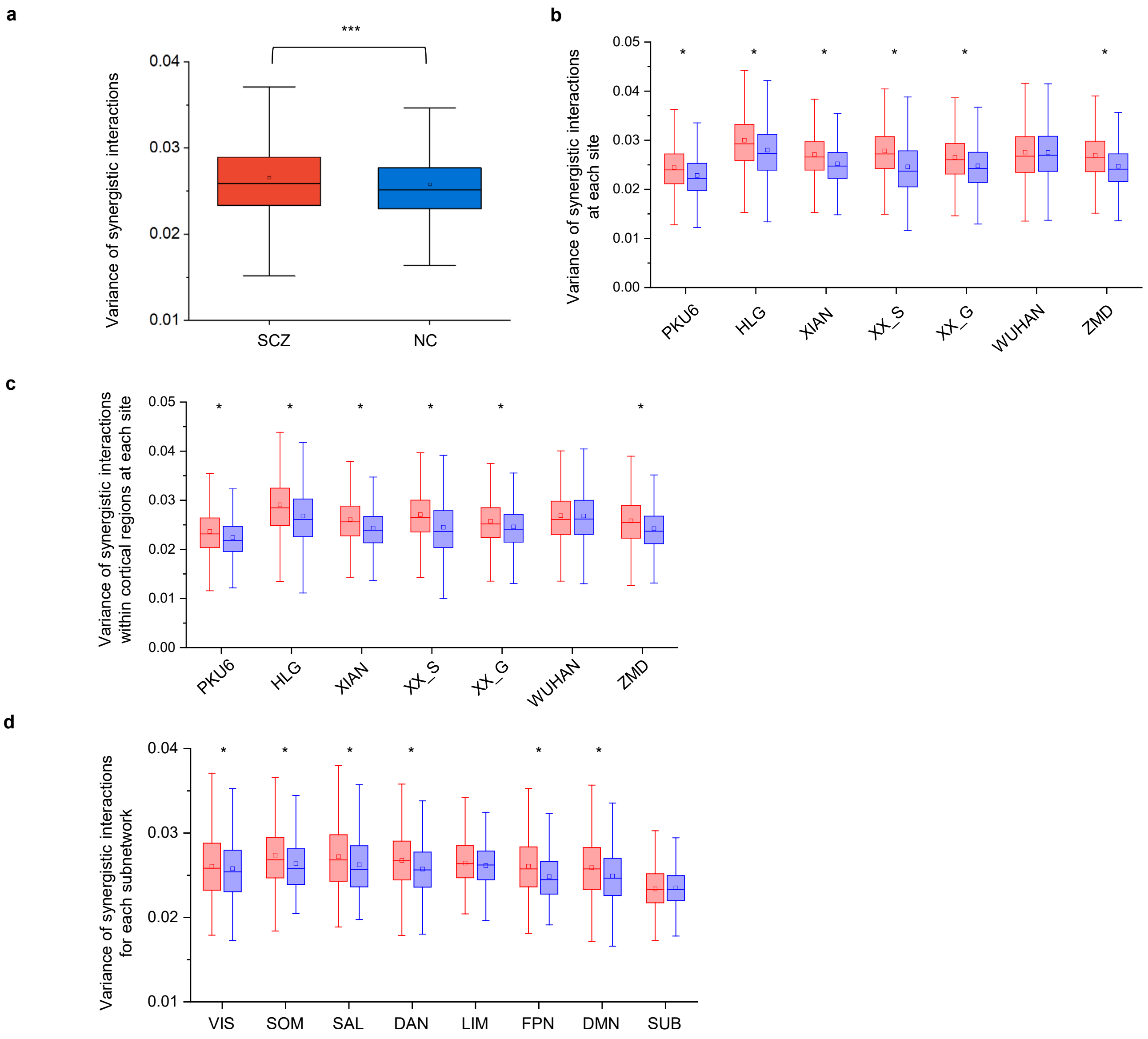

Figure S4. Synergistic interactions showed significantly higher inter-individual variance among SCZ compared to NC. a) Boxplot shows the variance in the distribution of synergistic interactions for SCZ and NC groups. Each point represents variance of a synergistic interaction. As indicated by the asterisks ($p < 0.001$; *t*-test), the variance of the synergistic interactions was significantly higher in SCZ group, compared to NC group. b) Higher variance of the synergistic interactions among SCZ compared to NC was significant at six of the seven sites. Items marked by asterisks indicate $p < 0.05$, FDR adjusted. At WUHAN site, the synergy variance of SCZ group (0.269±0.055) was slightly higher than that of SCZ group (0.268±0.054), although not reaching significance ($p = 0.57$). c) Higher variance of the synergistic interactions within cortical regions among SCZ compared to NC was significant at six of the seven sites, consistent with the results of considering the whole-brain synergy in b. Items marked by asterisks indicate $p < 0.05$, FDR adjusted. At WUHAN site, the synergy variance of SCZ group (0.274±0.058) was higher than that of SCZ group (0.272±0.056), although not reaching significance ($p = 0.11$). d) Higher variance of the synergistic interactions among SCZ compared to NC was significant for six of the eight networks. Items marked by asterisks indicate $p < 0.05$, FDR adjusted.

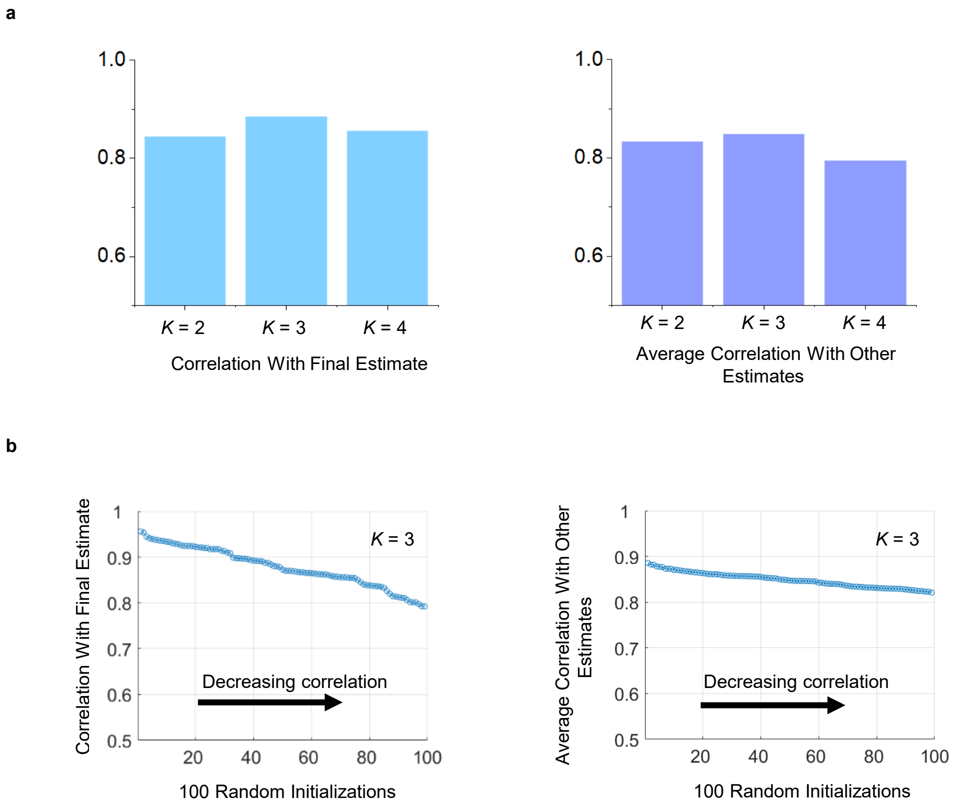

Figure S5. Correlations between the final estimate and solutions from the 100 random initializations in two-, three-, and four-factor estimates. a) We ran 100 times with random initializations for two-, three-, and four-factor estimates and we choosed the final estimate as solution having the highest average correlation with other solutions for each number of latent factors K. Factor estimation $K = 3$ achieved both the highest correlation with final estimate ($r = 0.89$, left) the highest average correlation with other estimates ($r = 0.84$, right), indicating that three-factor estimate was the most robust and stable answer. b) Scatter chart shows the distribution of correlations with final estimate (left) and the average correlation with other estimates (right) for $K = 3$.

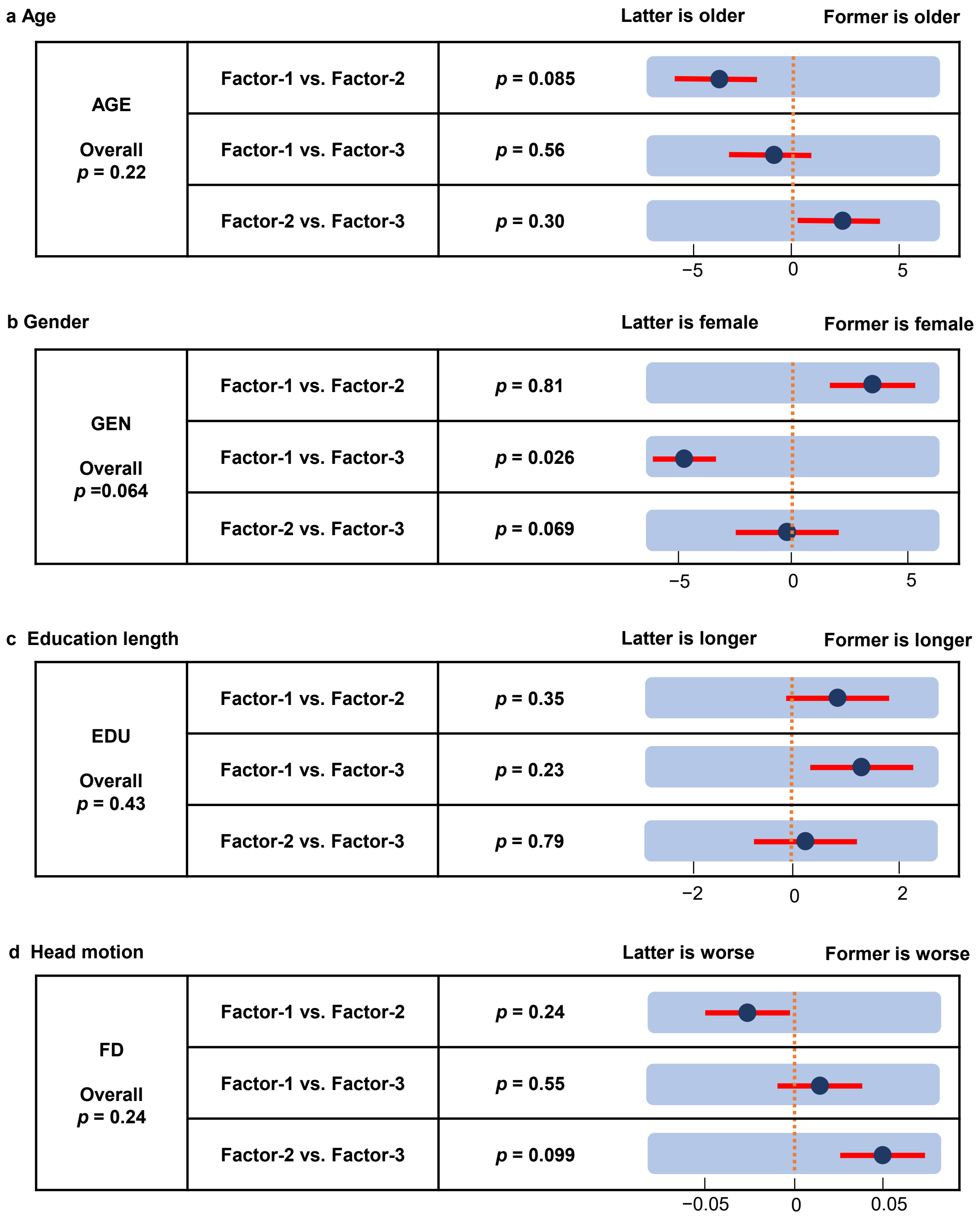

Figure S6. Differences in age, gender, education length and head motion across factors. No significant pairwise comparison remained after false discovery rate (FDR; $q < 0.05$) multiple comparisons correction. Blue dots are estimated differences between factors, and red bars correspond to standard errors. The result further validated the conclusion in Figure 3, demonstrating that LDA method reduced the correlation between factors and variables common to both NC and SCZ participants.

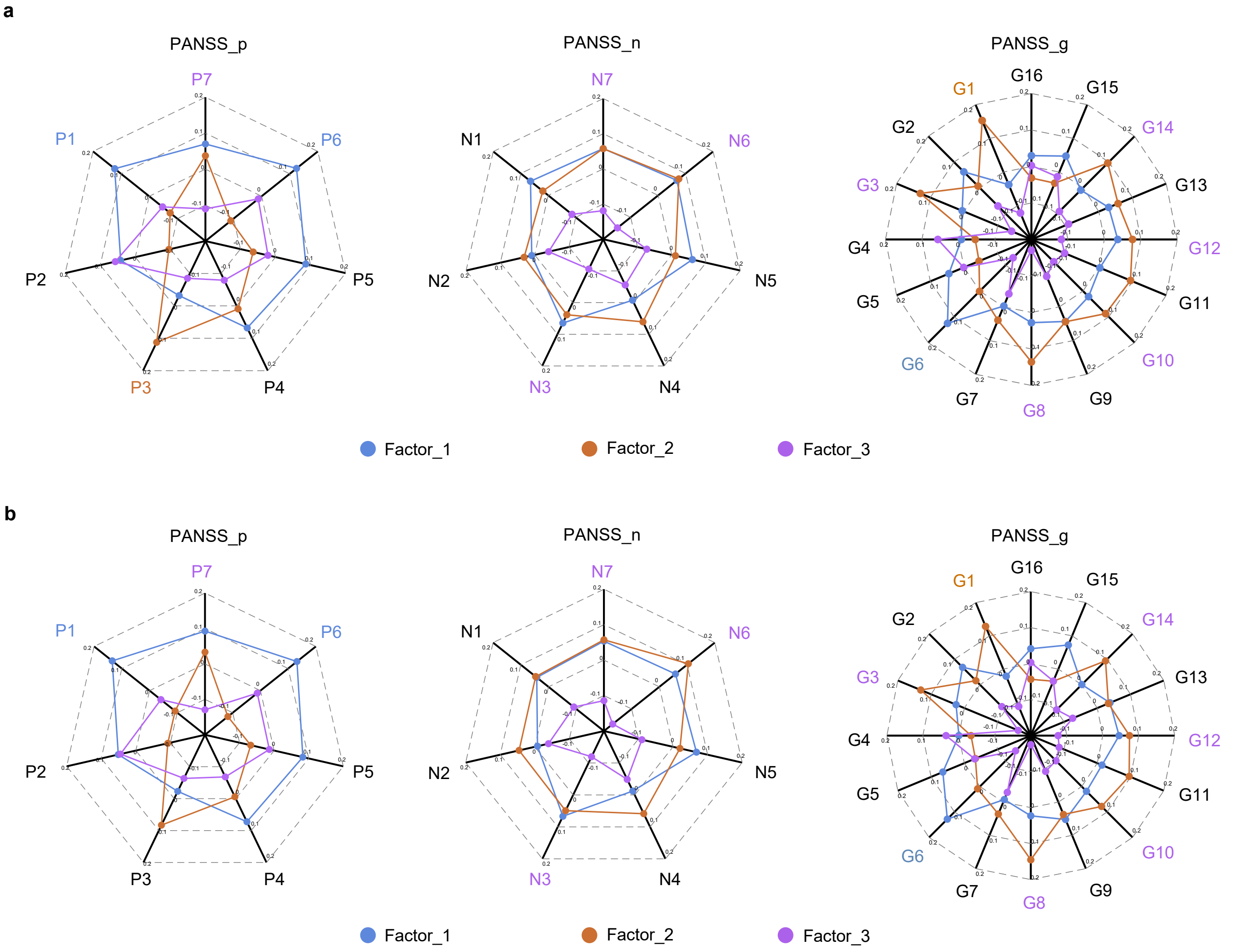

Figure S7. Associations between synergy factors and symptoms. a) Spider plot for Pearson correlations between the three latent factors and the 30 PANSS item scores, with control variables (site, gender, age) regressed out. Items marked in factor-specific colors indicate $p < 0.05$, FDR adjusted. Among them, 14 items (significantly more than the random value) showed a significant correlation with one of the factors. For positive subscale, blue line encloses the largest area, suggesting that factor 1 was positively correlated with most of these symptom items. For negative and general subscales, purple line encloses the smallest area, suggesting that factor 3 was negatively correlated with most of these symptom items. b) Spider plot for Pearson correlations of the 30 PANSS item scores with the three latent factors without regressing out control variables.

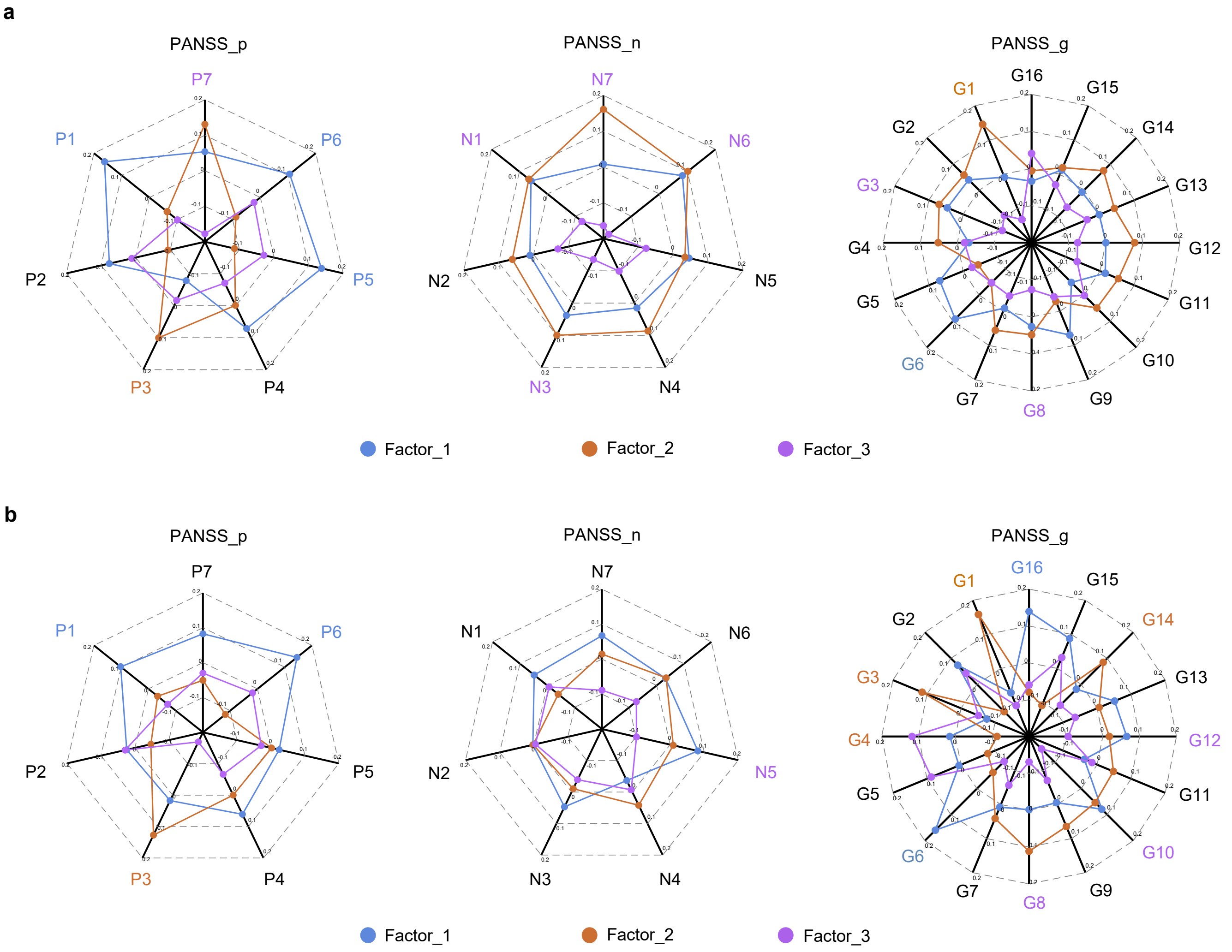

Figure S8. Associations between SCZ synergy factors and clinic symptoms in split-half control analysis. a, b) Spider plot for Pearson correlations of the 30 PANSS item scores with the three latent factors inferred by a) SCZ-Siemens ($N = 331$; sites: PKU6, HLG, XIAN, XX_S; scanner: Siemens) and b) SCZ-GE ($N = 207$; sites: XX_G, WUHAN, ZMD; scanner: GE). Items marked in factor-specific colors indicate $p < 0.05$, FDR adjusted.

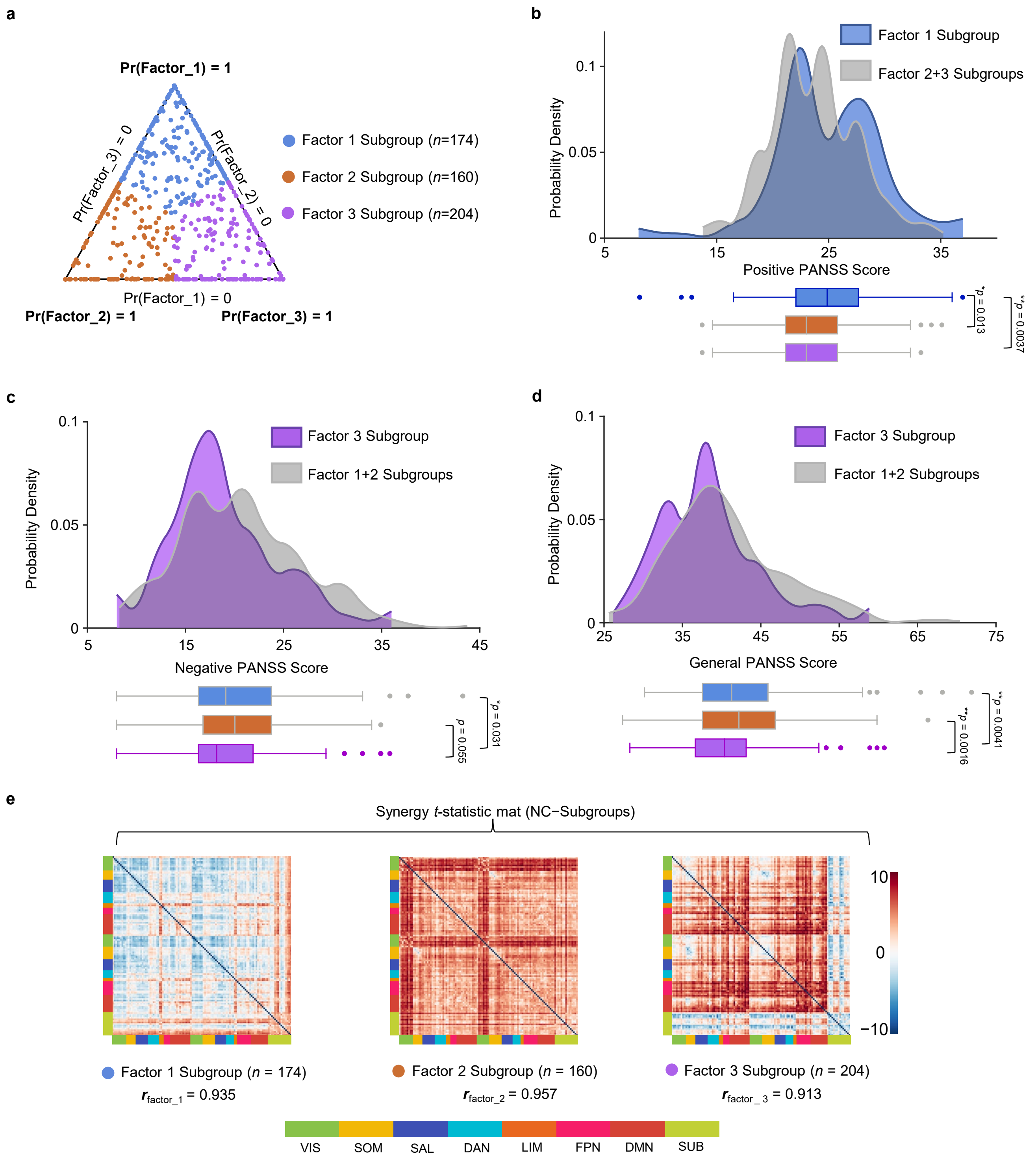

Figure S9. Three SCZ subgroups stratified based on factor compositions and between-group differences in PANSS scores. a) Each patient with schizophrenia was stratified into the corresponding subgroup according to the highest expression of synergy factors. Each dot corresponds to a participant, and its location in barycentric coordinates indicates this participant's factor composition. b) Factor 1 subgroup exhibited significantly higher positive PANSS scores. c) Factor 3 subgroup exhibited lower negative general PANSS scores. d) Factor 3 subgroup exhibited significantly lower general PANSS scores. e) The *t*-statistic mat (NC−Subgroups) of the synergy subgroups exhibited a similar connection pattern to the original synergy factors.

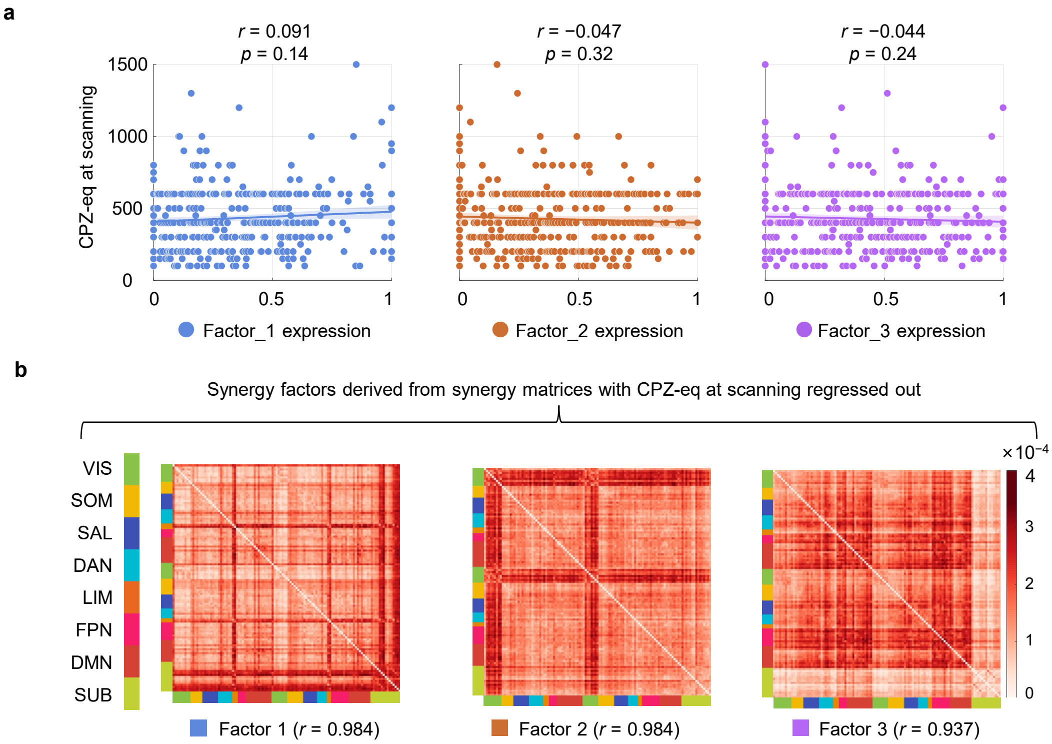

Figure S10. The relationship between the SCZ synergy factors and antipsychotic medication at scanning. a) Scatter plots of the association between chlorpromazine equivalent (CPZ-eq) at the scanning and three factor expressions for the medicated SCZ group. Correlation analyses were performed using Pearson's correlation. No significant correlation was observed (factor 1: r = 0.091, p = 0.014; factor 2: r = −0.047, p = 0.32; factor 3: r = −0.044, p = 0.24). b) Re-estimated factor-specific synergy patterns with CPZ-eq regressed out from each synergistic interaction for the synergy matrix of the medicated SCZ group. The patterns of the three factors are highly correlated with those of the original factors. (mean Pearson's correlation: r = 0.968).

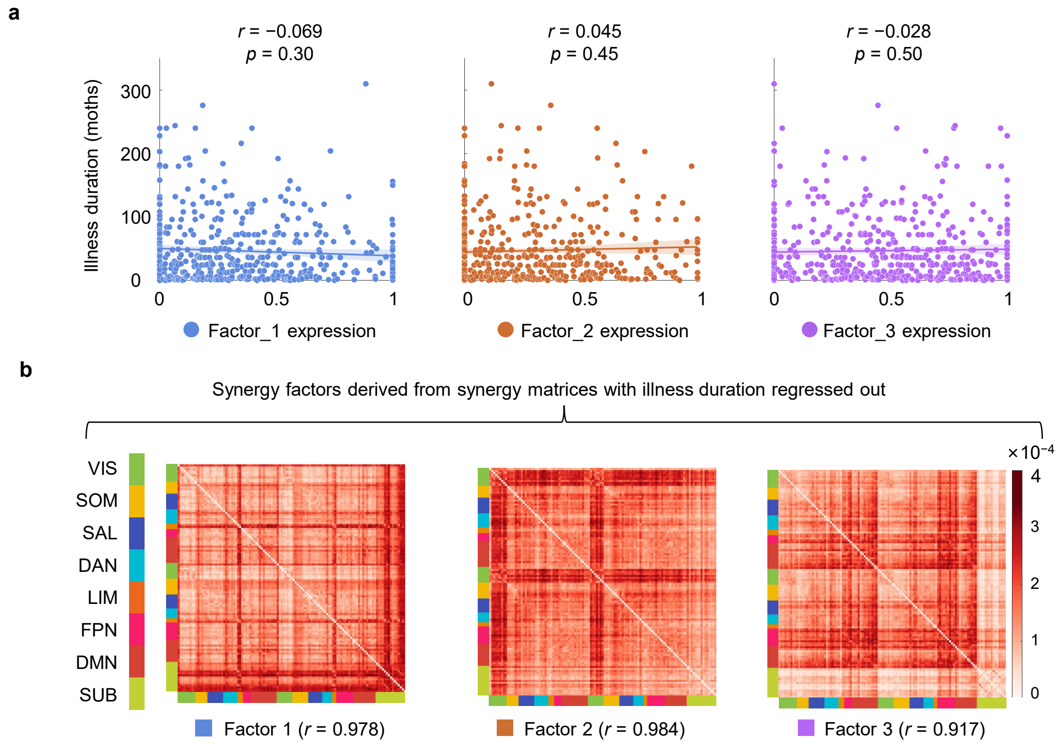

Figure S11. The relationship between the SCZ synergy factors and illness duration. a) Scatter plots of the association between illness duration (months) and three factor expressions for the SCZ patients. Correlation analyses were performed using Pearson's correlation. No significant correlation was observed (factor 1: $r = -0.069$, $p = 0.30$; factor 2: $r = 0.045$, $p = 0.45$; factor 3: $r = -0.028$, $p = 0.50$). b) Re-estimated factor-specific synergy patterns with illness duration regressed out from each synergistic interaction for the synergy matrix of the SCZ patients. The patterns of the three factors are highly correlated with those of the original factors. (mean Pearson's correlation: $r = 0.961$).

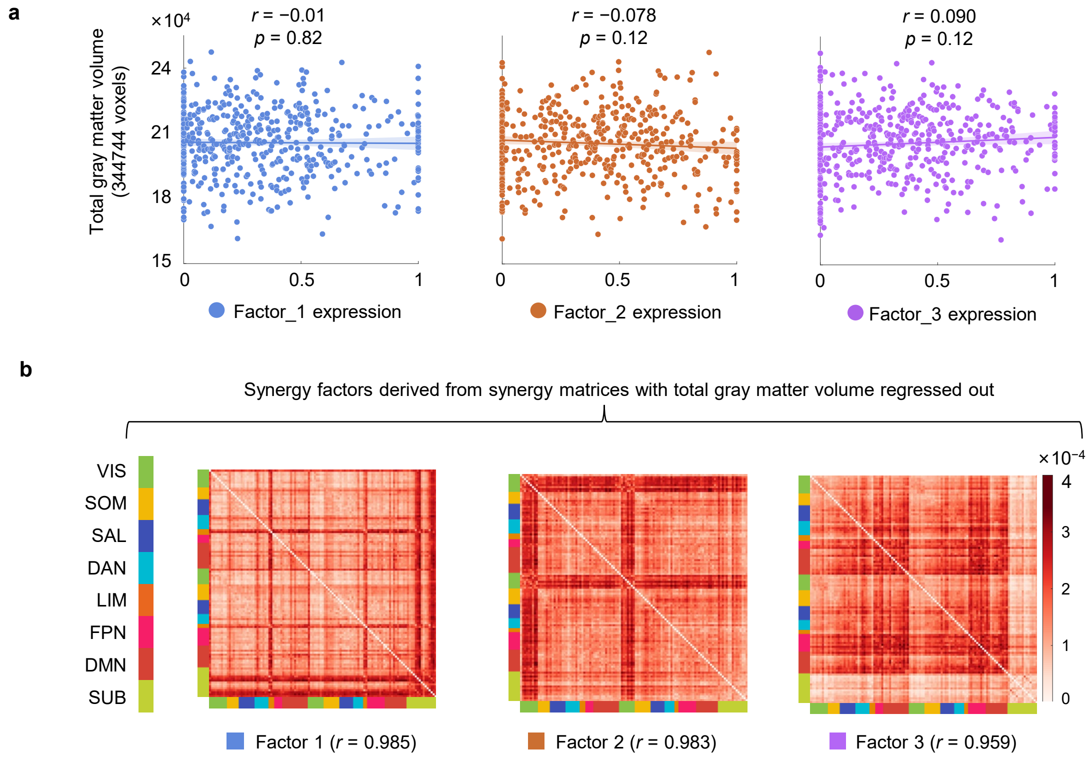

Figure S12. The relationship between the SCZ synergy factors and total gray matter volume. a) Scatter plots of the association between total gray matter volume and three factor expressions for the SCZ group. Correlation analyses were performed using Pearson's correlation. No significant correlation after FDR correction for multiple comparisons was observed (factor 1: $r = -0.01$, $p = 0.82$; factor 2: $r = -0.078$, $p = 0.12$; factor 3: $r = 0.09$, $p = 0.12$). b) Re-estimated factor-specific synergy patterns with total gray matter volume regressed out from each synergistic interaction for the synergy matrix of the SCZ group. The patterns of the three factors are highly correlated with those of the original factors. (mean Pearson's correlation: $r = 0.976$).

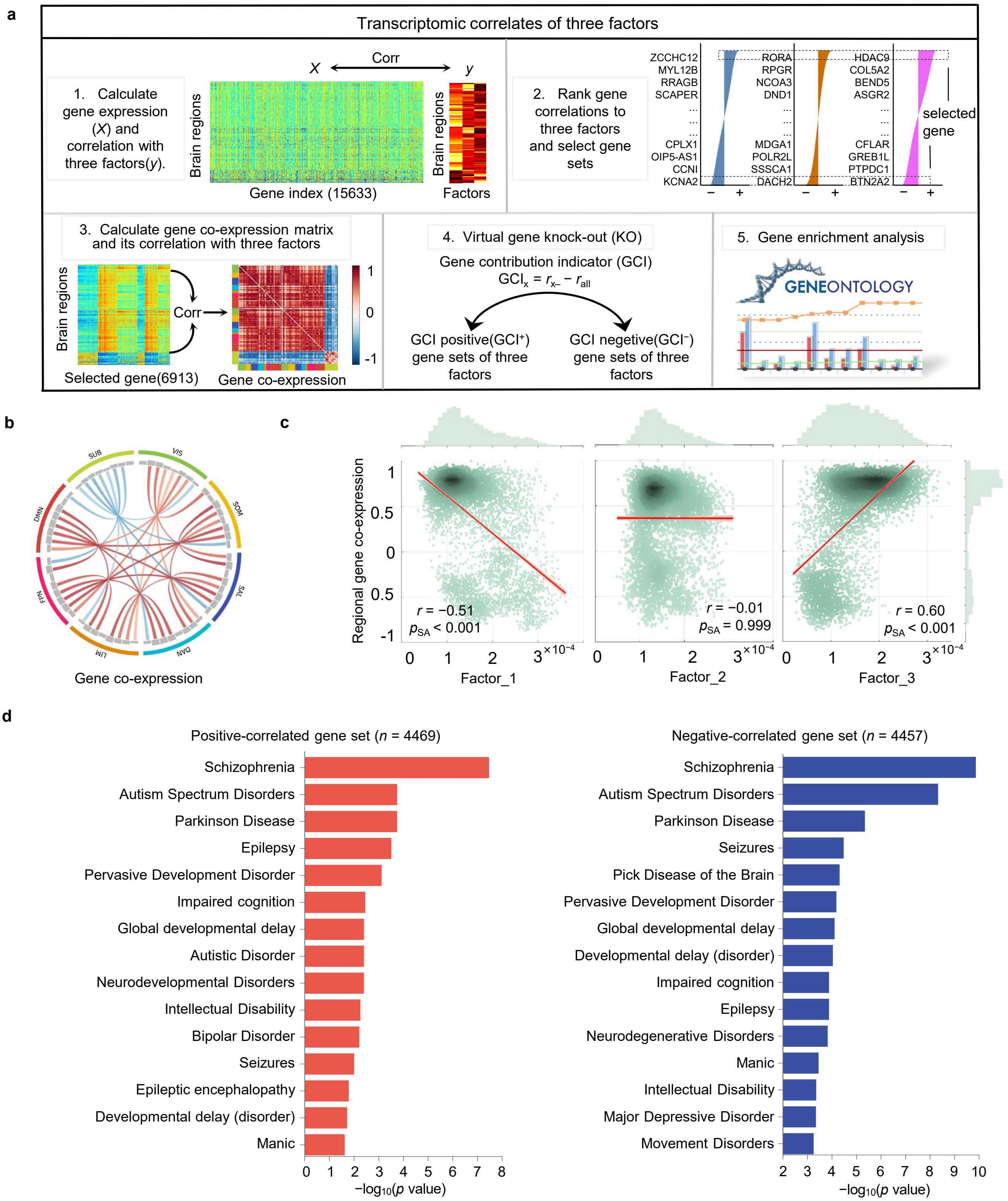

Figure S13. Transcriptomic correlates of synergistic interaction dysfunction patterns in three schizophrenia latent factors. a) Schematic of transcriptomics analysis to test whether gene expression explains three latent factors. First, we calculated Pearson correlations between gene expression at each brain region (ROI) and synergistic interaction dysfunction summed over ROIs for each factor. Second, we ranked gene by correlation coefficient, and selected top and bottom 10% for each factor as the factor-specific gene sets. Third, we calculated the gene expression similarity using the selected gene set and tested whether the constructed co-expression matrix correlates to the latent factors. Forth, we derived gene contribution indicator (GCI) gene sets using the virtual gene knock-out (KO) method.[6] Finally, we conducted GO enrichment analysis for GCI^+ and GCI^- gene sets of each factor. b) Chord plot shows gene co-expression matrix in a.3 averaged over the networks. The color intensity of chords is the same as the color bar in a.3. c) Gene co-expression correlates with the latent factors. The significance was estimated by a spatial autocorrelation (SA) permutation (spin of the gene expression) test. d) Bar plots showing the result of disease enrichment analysis for both of the top and bottom 10% factor-specific gene sets. The bar length indicates the p values (Bonferroni corrected). The result showed that the both of the top and bottom gene sets were most significantly enriched in the schizophrenia, which further confirmed three factors inferred by LDA as schizophrenia-specific factors.

a

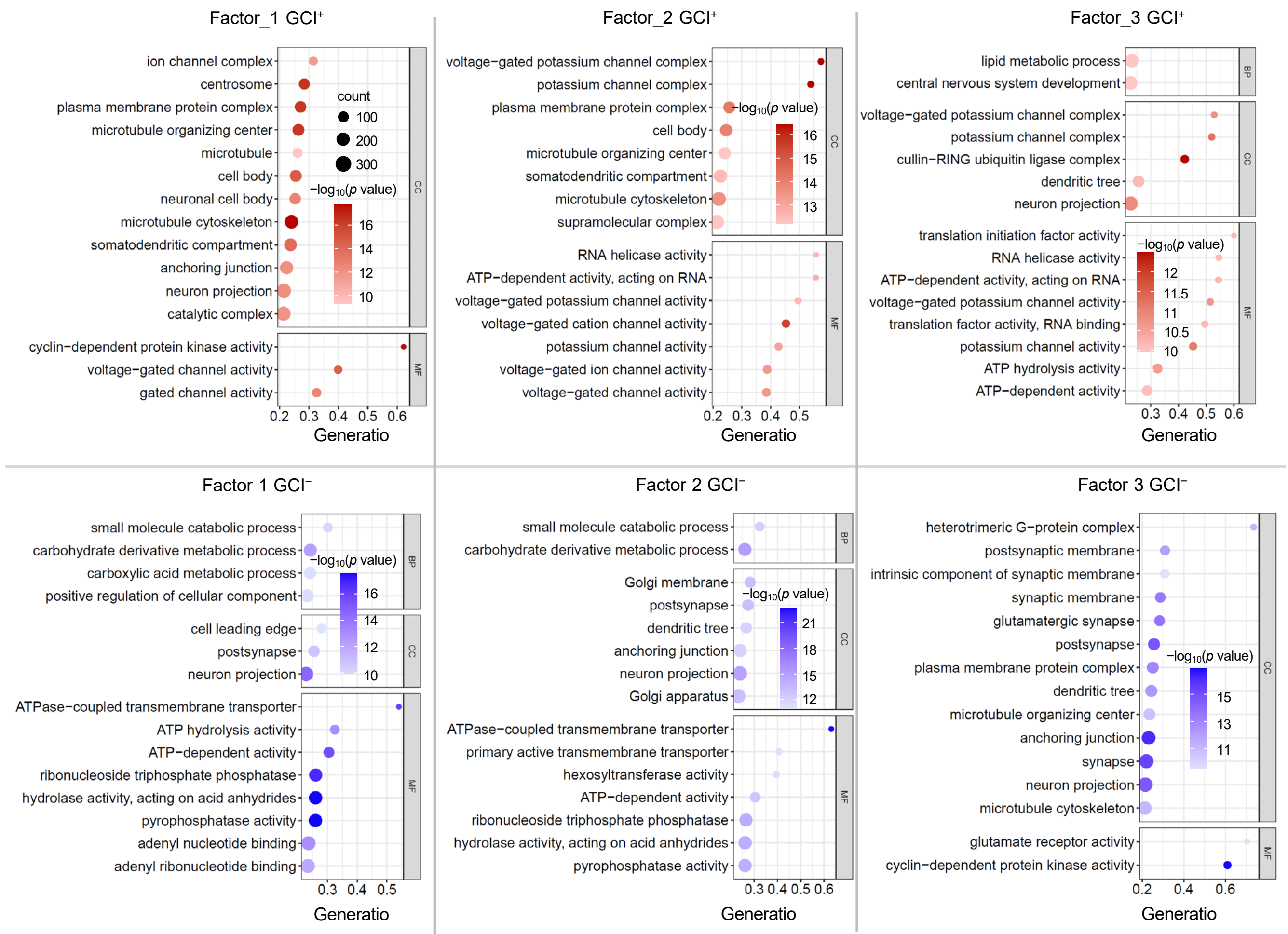

b

Factors	GO enrichment results: Range	GO enrichment results: Items
**Factor 1 (unique)**		**Fundamental neuron anatomical entity and cellular process:** centrosome; neuronal cell body; somatodendritic compartment; catalytic complex; hydrolase activity, acting on acid anhydrides; carbohydrate derivative metabolic process; pyrophosphatase activity; adenyl nucleotide binding; cyclin-dependent protein kinase activity
**Factor 3 (unique)**		**Neurotransmitter transport and signaling transduction:** synapse; synaptic membrane; glutamatergic synapse; potassium channel complex; plasma membrane protein complex; postsynaptic membrane; glutamate receptor activity
**Factor 1 ∩ Factor 2 ∩ Factor 3**		**Fundamental neuron components and functions:** postsynapse; neuron projection; plasma membrane protein complex; microtubule organizing center; microtubule cytoskeleton; ATP hydrolysis activity; ATP-dependent activity; ATPase-coupled transmembrane transporter activity

c

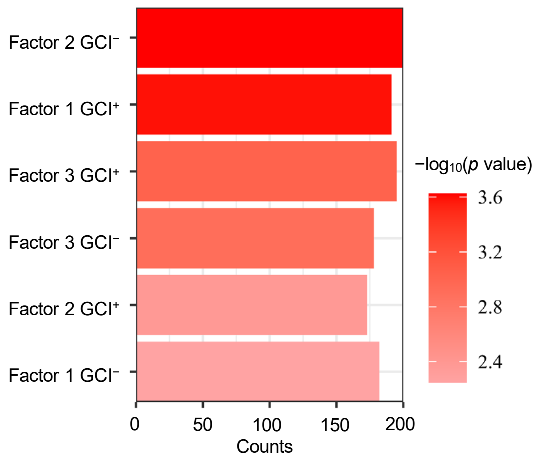

Figure S14. Validation analysis for three schizophrenia-specific factors from gene perspective. a) The gene ontology (GO) enrichment analysis for GCI$^+$ and GCI$^-$ gene sets of each factor. A Bubble plots showing the GO enrichment top 15 terms for GCI$^+$ (up) and GCI$^-$ (down). The biological process (BP), cellular component (CC), and molecular function (MF) are displayed separately. The dot size (count) represents the number of genes that are within the interest GCI$^+$ or GCI$^-$ gene panels as well as a specific GO term (y-axis). The different color intensities indicate the p values (Bonferroni corrected). b) Differences and similarities in GO enrichment results of factors. Factors 1 and 3 were the result of the most distinct pathological mechanisms. While factor 2 shared some similarities with both factors 1 and 3. c) 6 groups of GCI$^+$ and GCI$^-$ gene sets for each factor were all significantly enriched in the schizophrenia (Bonferroni corrected).

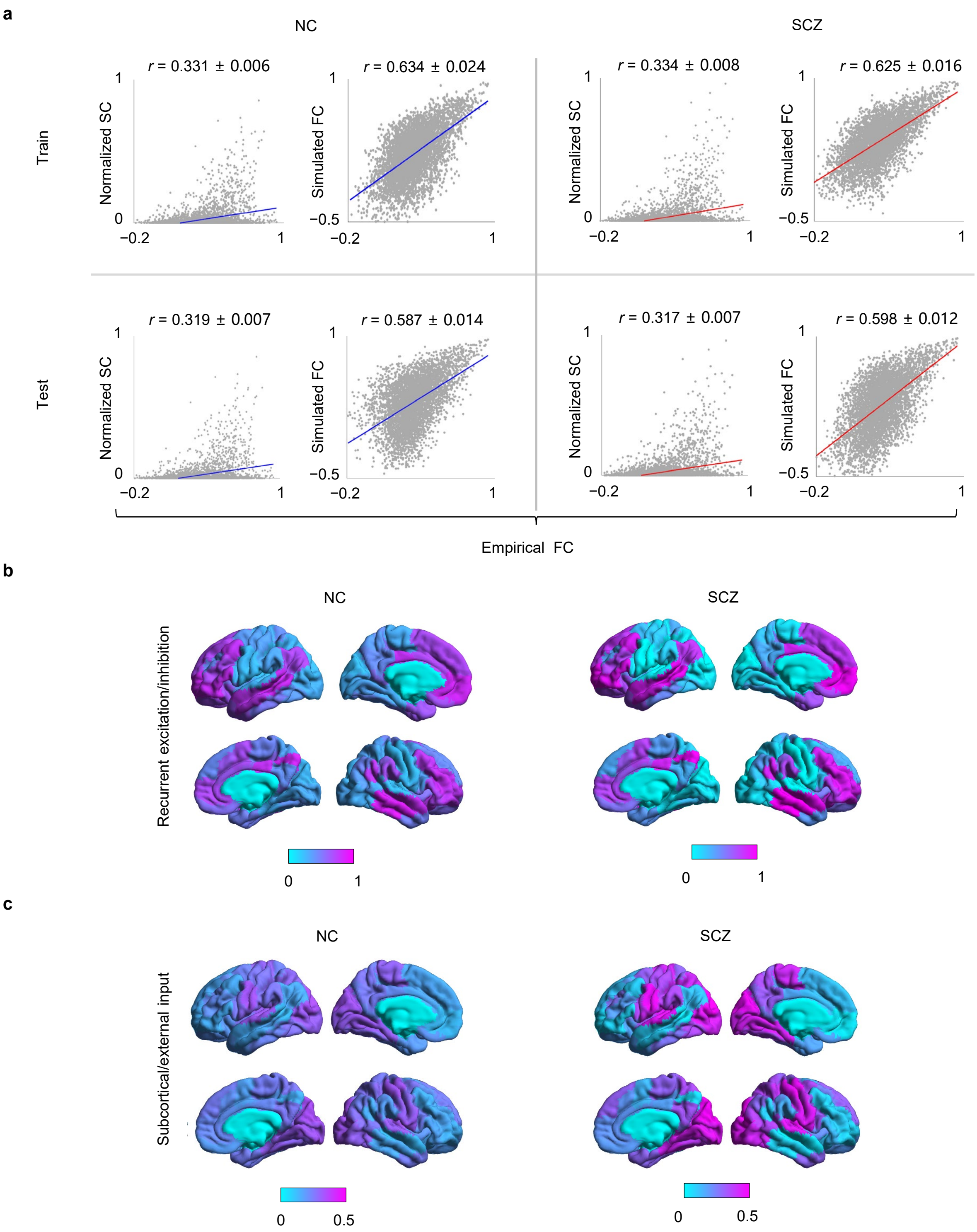

Figure S15. Microcircuit parameters and biophysical simulations. a) The scatter plot for linear correlations between empirical FC and SC, and empirical FC and simulated FC. Left: NC; right: SZ. Upper: train; lower: test. The optimal model predicted functional connectivity nominally higher than the corresponding baseline correlations between structural (SC) and functional connectivity (FC). b) Recurrent connection of NC (left) and SCZ (right) inferred from pMFM. c) Subcortical input of NC (left) and SCZ (right) inferred from pMFM.[7]

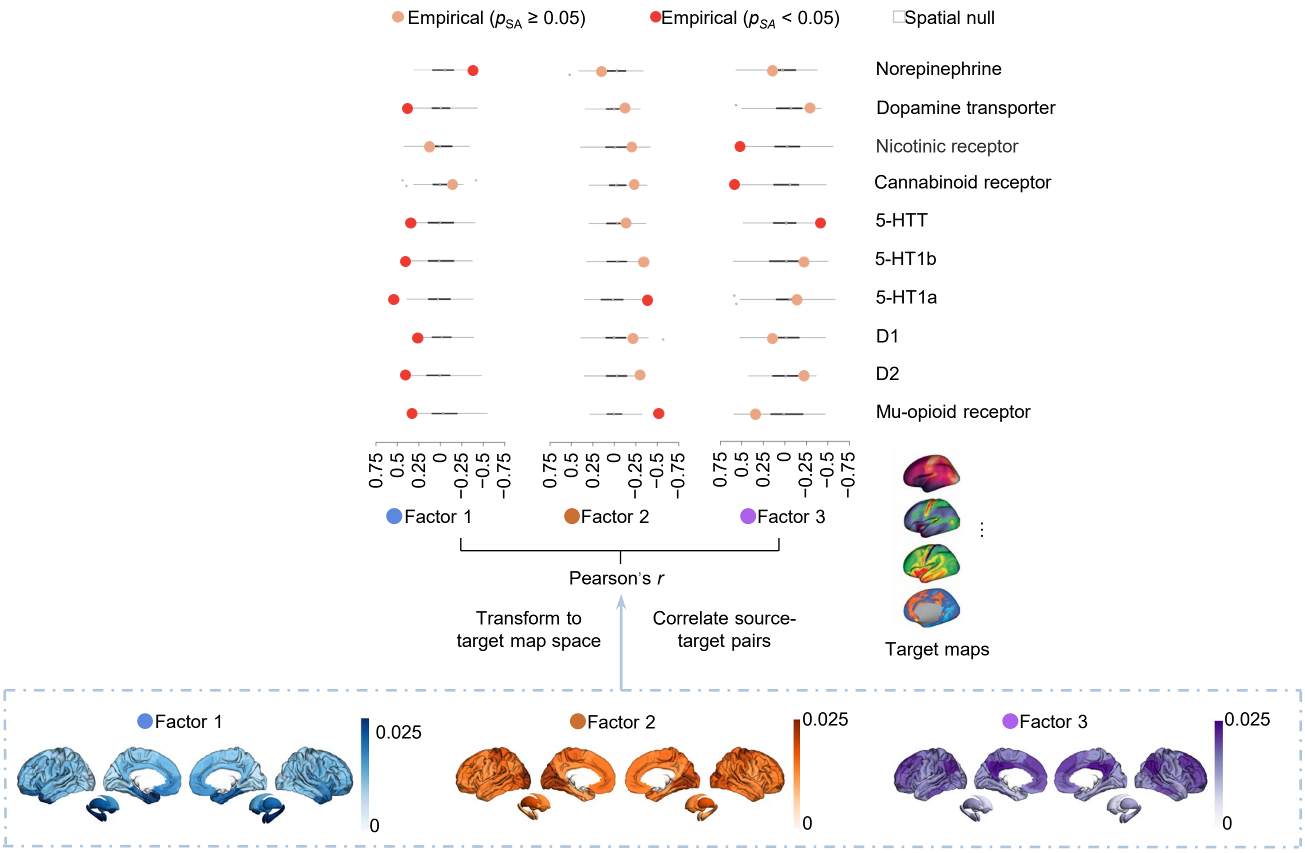

Figure S16. Application of Neuromaps to contextualize three synergy factor maps.[8] The factor maps were transformed into the native space of the target maps. Points represent Pearson's correlations between source and target maps (with significance defined as PSA<0.05). All correlations underwent correction for multiple comparisons.

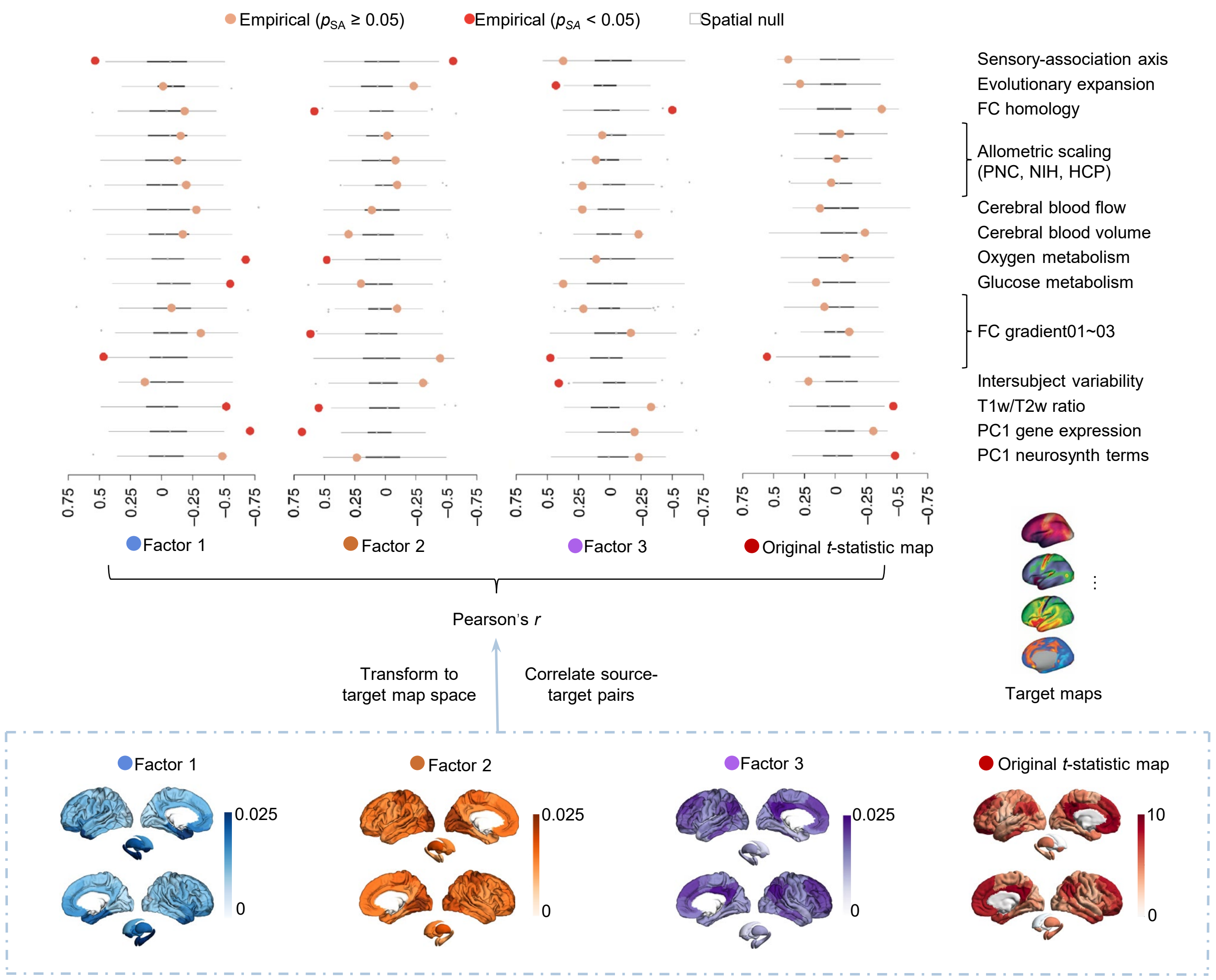

Figure S17. Use of Neuromaps to contextualize brain maps of the three factors. The analysis incorporated the classic 17 maps, which provide comprehensive insights into various aspects of neuroanatomy and function, offering in-depth details for the study of the spatial associations with the three factors. The findings revealed that these three factors corresponded to a diverse and distinct set of maps compared to the initial map of synergy dysfunction. This discovery sheds further light on the heterogeneity of schizophrenia and offers insights into potential mechanisms underlying its development.

Reference

[1] V. D. Calhoun, J. Sui, K. Kiehl, J. Turner, E. Allen, G. Pearlson, *Front. Psychiatry* **2012**, *2*, 75.

[2] R. F. Asarnow, K. H. Nuechterlein, D. Fogelson, K. L. Subotnik, D. A. Payne, A. T. Russell, J. Asamen, H. Kuppinger, K. S. Kendler, *Arch. Gen. Psychiatry* **2001**, *58* (6), 581.

[3] B. T. Yeo, F. M. Krienen, J. Sepulcre, M. R. Sabuncu, D. Lashkari, M. Hollinshead, J. L. Roffman, J. W. Smoller, L. Zöllei, J. R. Polimeni, *J. Neurophysiol.* **2011**,

[4] R. S. Desikan, F. Ségonne, B. Fischl, B. T. Quinn, B. C. Dickerson, D. Blacker, R. L. Buckner, A. M. Dale, R. P. Maguire, B. T. Hyman, *Neuroimage* **2006**, *31* (3), 968.

[5] L. Fan, H. Li, J. Zhuo, Y. Zhang, J. Wang, L. Chen, Z. Yang, C. Chu, S. Xie, A. R. Laird, *Cereb. Cortex* **2016**, *26* (8), 3508.

[6] J. Seidlitz, F. Váša, M. Shinn, R. Romero-Garcia, K. J. Whitaker, P. E. Vértes, K. Wagstyl, P. K. Reardon, L. Clasen, S. iu, *Neuron* **2018**, *97* (1), 231.

[7] X. Kong, R. Kong, C. Orban, P. Wang, S. Zhang, K. Anderson, A. Holmes, J. D. Murray, G. Deco, M. van den Heuvel, *Nat. Commun.* **2021**, *12* (1), 6373.

[8] R. D. Markello, J. Y. Hansen, Z.-Q. Liu, V. Bazinet, G. Shafiei, L. E. Suárez, N. Blostein, J. Seidlitz, S. Baillet, T. D. Satterthwaite, *Nat. Methods* **2022**, *19* (11), 1472.
